# Should we really use graph neural networks for transcriptomic prediction?

**DOI:** 10.1093/bib/bbae027

**Published:** 2024-02-12

**Authors:** Céline Brouard, Raphaël Mourad, Nathalie Vialaneix

**Affiliations:** Université Fédérale de Toulouse, INRAE, MIAT, 31326 Castanet-Tolosan, France; Université Fédérale de Toulouse, INRAE, MIAT, 31326 Castanet-Tolosan, France; Université Paul Sabatier, 31062 Toulouse, France; Université Fédérale de Toulouse, INRAE, MIAT, 31326 Castanet-Tolosan, France

**Keywords:** deep learning, graph neural network, transcriptomic, phenotype prediction

## Abstract

The recent development of deep learning methods have undoubtedly led to great improvement in various machine learning tasks, especially in prediction tasks. This type of methods have also been adapted to answer various problems in bioinformatics, including automatic genome annotation, artificial genome generation or phenotype prediction. In particular, a specific type of deep learning method, called graph neural network (GNN) has repeatedly been reported as a good candidate to predict phenotypes from gene expression because its ability to embed information on gene regulation or co-expression through the use of a gene network. However, up to date, no complete and reproducible benchmark has ever been performed to analyze the trade-off between cost and benefit of this approach compared to more standard (and simpler) machine learning methods. In this article, we provide such a benchmark, based on clear and comparable policies to evaluate the different methods on several datasets. Our conclusion is that GNN rarely provides a real improvement in prediction performance, especially when compared to the computation effort required by the methods. Our findings on a limited but controlled simulated dataset shows that this could be explained by the limited quality or predictive power of the input biological gene network itself.

## INTRODUCTION

Machine learning (ML) is increasingly used for transcriptomic-based predictions. For instance, in cancer, ML can be used to predict phenotypes such as the cancer type or the likelihood of a patient responding to a specific treatment [1, 2]. However, predicting phenotype from transcriptomic data is challenging due to the high dimensionality, the small-to-moderate sample size and the high variability between experiments. Classical ML methods, including logistic regression, support vector machine, Random Forest and more recently deep learning methods, have been proposed to tackle this question [3].

In cells, genes are organized into regulatory networks that consist of sets of genes, or parts of genes, that interact with each other to control specific cell functions. Therefore, various works have advocated for the use of gene network information in ML models to improve the performance of phenotype predictions [4, 5]. In this scope, graph neural networks (GNNs) were recently proposed: GNN is a particular type of convolutional neural network (CNN) where a graph representing pairwise relationships between nodes is used to drive the convolution [6, 7]. Using biological knowledge on gene regulatory networks (through PPI networks or co-expression networks) in this type of models, some authors (e.g. [8, 9]) showed that this approach can outperform Random Forest and glmgraph [10] for metastatic event prediction.

However, in other fields of applications where GNN are widely used (chemistry or combinatorial optimization), recent works [11–13] tend to show that this type of models, if they sometimes improve the prediction performance, are also frequently over-complex for the task. A similar work [14] even showed that classical ML methods often outperform deep learning for phenotype prediction. Overall, the conclusion is that simpler models less demanding to train can obtain comparable results, which questions the ratio between benefits and costs (in particular computational and thus environmental cost) of these methods. This question is particularly relevant for phenotype prediction from gene networks, since the input graph is usually only a very crude proxy of the true underlying regulatory mechanism between genes: most used networks in GNN applications that we found are only PPI based networks, which are known to reflecting more an ensemble of likely networks than the real regulatory network [15, 16]. As for regulatory networks recovered from computational methods, [17] and [18] have shown that they are no better than random guess in some cases, especially when the regulatory mecanisms are complex, like in eukaryotes, and that the method is based on RNA levels only. In some eukaryotes, the mRNA levels of transcription factors and their targets has indeed been found to be low [17], which not only questions the relevance of network inference methods but also probably of using the regulatory network as a good addition to expression data for prediction.

In the current article, we present a comprehensive and reproducible benchmark comparing GNN to other ML methods for transcriptomic-based phenotype prediction. Our comparison uses previously published datasets and models that are systematically compared using a common ground methodology. Our main conclusion is that, as expected but rarely reported, GNNs have performances comparable to simpler methods. We point a few explanations for this conclusion, both related to the reliability of the input graph and to a more general reproductibility problem as already discussed in [19]. Overall, our hope if that this work helps to better account for the model complexity in evaluating the performance gain in future computational tool development.

## GNNS AND THEIR USE FOR PHENOTYPE PREDICTION

This section briefly describes the basic ground common to GNN methods and how they integrate gene regulatory information with gene expression to perform predictions.

The basic principle of GNN [20–23] is to extend the convolutional filters (or kernels) used in CNN to extract patterns and stationary properties from local (and structured) information in the data. More precisely, the graph edges are used as a way to obtain local information by the computation of convolutional filters. A general presentation of most convolution approaches used in GNN is described in [24] using a message passing framework: if $\mathcal{G} = (X,E)$ is a graph with $p$ nodes ($x \in X$) and edges $e \in E$, with (potentially missing) associated node and edge features $l _{x}$ and $l _{e}$, GNN iteratively learns a latent representation of node $x$ at step $t+1$ using a message passing approach of the form 


\begin{align*} h_{x}^{t+1} = F\left(h_{x}^{t}; {\square}_{y: (x,y) \in E} \phi_{t}(h_{x}^{t}; h_{y}^{t}; e_{(x,y)})\right), \end{align*}

where $\square $ is a given operator (usually $\square := \sum $) and $F$ and $\phi _{t}$ are parameterized functions which parameters are learned during the training. The initial representation is usually taken as $h^{0}_{x}:= l_{x}$ and the prediction is obtained as either $h^{T}_{x}$ (where $T$ is the last iteration) or as a global numeric value obtained after additional pooling layers that concatenate sets of $(h^{T}_{x})_{x \in \mathcal{S} \subset X}$. Several variants of this model have been made available in the Python libraries Spektral [25] (which we used for our simulations) and PyTorch [26].

In particular, in the remaining of this paper, we use the GNN “ChebNet”, proposed in [20], which is based on a spectral decomposition of the graph (through the graph Laplacian eigendecomposition) and Chebyshev polynomials to define localized filters. ChebNet also contains graph coarsening and pooling layers which perform iterative pooling operations using graph-based aggregations (called coarsening).

In our use of the method, $\mathcal{G}$ is a graph representing gene regulations, $X$ is the set of genes and $l_{x} \in \mathbb{R}$ is the expression of gene $x$ observed in one individual (one sample). Roughly, gene expression is used as the message passed from one gene to its regulating genes, which aims to mimic the biological process at hand in the cell. Finally, coarsening and pooling operations are used to aggregate the resulting graph signal, which is finally passed to a fully connected layer (or several fully connected layers) that handles the final prediction of the phenotype (a real number or a class $\{1, \ldots , M\}$).

## MATERIAL AND METHODS

### Data description

We selected datasets from a subset of articles for which a reasonable amount of information was available to reproduce experiments. More precisely, we selected articles for which the gene expression dataset was available with all pre-processing and filtering steps. Moreover, we selected datasets from articles for which the gene network was also provided with all pre-processing steps (if needed), and part of the source code for experiments. Used datasets are described in rows 1–4 of Table 1. Links for data and code availability are given in Table 2 when possible. Moreover, Supplementary Table S1 provides a more detailed description of the dataset characteristics and associated prediction tasks.

**Table 1:** Datasets’ description

Dataset	# nodes	# samples	Prediction type (# classes)
BreastCancer [8, 28]	6888	969	Classification (2)
CancerType [29]	4444$^{1}$	11,070	Classification (34)
F1000 prostate [9]$^{2}$	978	25,565	Classification (9)
F1000 full [9]	978	156,461	Classification (12, 14, 49)$^{3}$
Simulated (new)	21	100	Regression
DREAM5	1564	805	Regression

$^{1}$ The authors used different networks, resulting in different numbers of nodes. Here, we report the number of genes of the PPI network (used in our simulations) but this network was augmented by singleton nodes (up to $7091$) for GNN simulations, in the same way as the best model reported in [8]. $^{2}$Two different graphs were used for this dataset in [9], one that is tissue specific and the other that is not tissue specific and is common with ‘F1000 full’. Here, we report the results obtained with tissue independent network, found to be better for prediction in [9]. $^{3}$Three different prediction tasks were performed for outputs (primary site, subtype and MOA, respectively) with different numbers of classes [29].

**Table 2:** Datasets’ availability

Dataset	Expression	Network	GNN code	Folds	Hyper-parameter
	availability	availability	availability	availability	availability
BreastCancer [8, 28]	Yes$^{1,7}$ for [8]	Yes$^{1,7}$ for [8]	Yes$^{2}$ for [8]	No$^{7}$	In source code
CancerType [29]	Yes$^{3,7}$	Yes$^{3,7}$	Yes$^{4}$	Yes$^{3,7}$	In article and source code
F1000 [9]	From authors$^{7}$	From authors	Yes$^{5}$	From authors$^{7}$	Yes$^{6}$
Simulated (new)	Yes$^{8}$	Yes$^{8}$	Yes$^{9}$	Yes$^{8}$	Yes$^{9}$
DREAM5	Yes$^{10}$	Yes$^{10}$	Yes$^{8}$	Yes$^{8}$	Yes$^{9}$

$^{1}$ http://mypathsem.bioinf.med.uni-goettingen.de/resources/glrp [accessed 27 September 2022]. $^{2}$https://gitlab.gwdg.de/UKEBpublic/graph-lrp [accessed 13 October 2022]. $^{3}$https://drive.google.com/drive/folders/1_Cnvab7mIwCrNJyY-J4aR2ck9i72KH8t?usp=sharing [accessed 13 October 2022]. $^{4}$https://github.com/RicardoRamirez2020/GCN_Cancer [accessed 13 October 2022]. $^{5}$https://github.com/mmcdermott/cnn_graph [accessed 27 September 2022]. $^{6}$in article and in https://github.com/mmcdermott/LINCS_Deep_Learning_Benchmarks [accessed 27 September 2022]. $^{7}$some preprocessed files (format conversion, scaling, etc.) and additional networks (complete, random and patient random) have been made available at https://doi.org/10.57745/BZ0TTC [released 11 July 2023]. $^{8}$at https://doi.org/10.57745/BZ0TTC [released 11 July 2023]. $^{9}$at https://forgemia.inra.fr/nathalie.villa-vialaneix/gnn.git [released 20 September 2023 and 19 December 2023]. $^{10}$at https://www.synapse.org/#!Synapse:syn2787209 [accessed 8 November 2023] (training data / Network 1 - in silico/net1_expression_data.tsv and test data / DREAM5_NetworkInference_GoldStandard_Network1 - in silico.tsv

In addition, to provide positive control examples, we also used two simulated datasets. One was a small and well-controlled dataset generated with the simulation tool sismonr (https://oliviaab.github.io/sismonr/) [27]. More precisely, this dataset was simulated from 20 genes, with two copies of each gene. The probability of the genes to be protein-coding genes was set to 70% (as was done in the tool tutorial). Two hundred time steps were simulated for 100 independent individuals.

The second simulated dataset was obtained from a similar mechanistic model and previously made available through the DREAM5 challenge on network inference (https://dreamchallenges.org/dream-5-network-inference-challenge/). From this challenge, we downloaded on the wiki page *In silico* test (network) and training (static expression) data. We randomly chose one gene as the target gene expression to predict from the other gene expressions and the network. Gene expressions were previously centered and scaled.

For some of the datasets, additional preprocessings were sometimes performed compared to the published (BreastCancer) or generated (Simulated) datasets, in line with the original code from authors. These preprocessings are fully described in Section 1.2 of Supplementary material.

All resulting (raw and preprocessed) datasets that were not available elsewhere, as well as the source code that generated them, the computing environment information (including R and package versions) and a basic exploration of the results are available in our persistent data repository https://doi.org/10.57745/BZ0TTC and in our source code repository https://forgemia.inra.fr/nathalie.villa-vialaneix/gnn.git.

### Methods and hyper-parameters

As a baseline for comparison between GNN and simple methods, we used standard ML methods, already tested in most of the original articles. This included Random Forest, multilayer perceptron and SVM (or its regression version, SVR). In some cases, whenever the dataset size allowed it, we also trained a concurrent graph based approach, glmgraph [10]. glmgraph is a graph-constrained regression model, either linear regression or logistic regression (The method is available as an R package that was archived on CRAN on 4 July 2021 but can still be installed from archived source package.). Finally, to better assess the potential of GNN methods, we also implemented a GNN approach based on convolution between observations rather than between features. This approach was inspired by the successful application of GNNs for node predictions (such as documents) in a graph (such as a citation network), where convolutions are applied to node representions [22]. These methods will be denoted, respectively, by RF, MLP, SVM, glmgraph and GNNo in the sequel. Table 3 gives a comparison of methods tested and discussed in the different articles versus what we implemented for each dataset.

**Table 3:** ML methods tested in the original articles (as given in Table 2) and in the current article

Dataset	Tested	Code	Tested
	in paper	availability	here
BreastCancer	RF, MLP, glmgraph	No	RF, SVM, MLP, glmgraph, GNNo
CancerType		NC	RF, SVM, MLP, GNNo
F1000	RF, MLP$^{1}$	No$^{2}$	RF, SVM, MLP, GNNo
Simulated	NC	NC	RF, SVM, MLP, glmgraph, GNNo
DREAM5	NC	NC	RF, SVM, MLP, glmgraph, GNNo

$^{1}$ Also, $k$-nearest neighbors, classification trees and linear classifiers. $^{2}$but tuned hyper-parameters are provided in their repository.

To avoid as much as possible data leaking, we systematically used cross-validation (CV) and reported results based on the test datasets. CV folds were chosen identical to those by the authors when available (CancerType, F1000) or were generated randomly and used in all experiments otherwise (BreastCancer, Simulated, DREAM5). They are made available in our data and code repositories. In addition, the following policy was used for the choice of hyper-parameters:

- For GNN, we systematically used hyper-parameters (and code) provided by the authors of the original paper (from which we extracted the dataset; see Table 2). Most of the time, the method was run on the graph reduced to isolated nodes (unless performed otherwise by the authors).
- for RF, MLP and SVM, we used the default hyper-parameters of the used function/library, except for RF (taken from scikit-learn [30]) for which we increased the number of trees in the forest from 100 to 500 (because, according to our personal experience of the method, 100 is usually not enough to properly train the method and is not the default of other implementations like the seminal randomForest R package). Note that purposely not tuning hyper-parameters of these methods is expected to penalize their performance compared to GNN where hyper-parameter tuning has very likely been performed by the authors of the original paper. In addition, for F1000, [9] have performed a careful hyper-parameter tuning where a 10-fold CV was used with a fold to compute the cross-validation error, a fold to compute the validation error and the hyper-parameters were set so as to minimize the overall validation error. For the sake of homogeneity with the other datasets, we only excluded one fold out of the training for computing the cross-validation error, both with default function hyper-parameters and with their tuned hyper-parameters for comparison. This can explain slight differences between their published results and ours. Also, in this very specific case, our results with their published hyper-parameters thus contain a very mild data leaking issue.
- For glmgraph, since the regularization parameter strongly impacts the results, we proceeded as described in [28] by tuning it. However, to avoid data leaking, we added an additional 5-fold CV loop nested in the original CV loop to fairly choose this value in each training fold (which increased the already high computational cost for this method).
- For GNNo, we used the same hyper-parameters as for the MLP implementation. The graph was estimated by computing pairwise Euclidean distances between patients/individuals and by keeping only the 0.2$\%$ lowest distances as edges in the graph.

The precise functions and values of the hyper-parameters for each experiment is provided in our code repository and in Tables S2–S5 of our Supplementary material.

### Assessing the impact of the input graph, the GNN type and the implementation

Various additional questions are also addressed by including some variations to the baseline simulation framework described in the previous section.

#### Impact of the implementation

For GNN, we used the implementation provided by [8] on all datasets. This implementation is based on the one initially provided by [20]. More precisely, we kept the coarsening approach as provided in the original code and re-implemented (for the sake of clarity) the GNN model based on the Spektral [25] and keras (https://keras.io) libraries. We made sure that this simplification of the code did not change the obtained results. On CancerType, we also compared this approach to the original implementation of [29] that slightly differs from the previous one for the choice of the order in Chebyshev convolution (fixed to 1 in the later, thus equivalent to GCN [22]).

For GNNo, Spektral could not be used (because the batch sizes can not be customized when using a convolution between individuals in this library). Hence, we slightly modified the implementation of GNN from keras (https://keras.io/examples/graph/gnn_citations/) instead, by keeping only one graph convolutional layer instead of two.

Finally, the impact of the implementation was also assessed by running the same methods with different implementations. More precisely:

- Multilayer perceptron was implemented using the function included in the Python libraries scikit-learn [30] and keras/tensorflow 2.
- SVM was implemented using the functions included in the Python library scikit-learn and in the R package e1071. Both implementations are based on the C++ library LIBSVM [31].
- Random Forests were implemented using the functions included in the Python library scikit-learn and in the R package randomForest [32].

#### Impact of the input graph

To challenge the usefulness of the added information in graph-based models (GNN and glmgraph), we also used the exact same methods with different naive graphs for the dataset BreastCancer. More precisely, we trained the same model with the following:

- A very basic graph obtained from a simple thresholding of the Pearson correlation matrix between genes. These results are named ‘Cor’ in the sequel.
- Two dummy graphs unrelated to biological information: in the first, we used the configuration model to obtain one random graph, sampled uniformly at random among the random graphs with the same degree distribution as the original graph [33, 34]. This model consists in performing a large enough number of random permutations between gene edges in the original graph. The results related to this graph are named ‘random’. In addition, a complete graph is also used in place of the original graph. Results associated to this graph are termed ‘complete’ in the sequel.

We chose to test this variation on BreastCancer only for the sake of simplicity, to avoid multiplying the sources of variations and the diversity of results. BreastCancer is the most adequate dataset for this comparison since its relatively moderate size allowed for the computation of all graph-based methods (glmgraph, GNN and GNNo) on the different graphs (some methods, like glmgraph, were too extensive to be used with CancerType and with some F1000 datasets). Also note that BreastCancer is one dataset that answers a biologically relevant question (metastatic event prediction, which is a difficult medical question) and that it exhibits contrastive performances across methods, contrary to CancerType (see Results and Discussion).

#### Impact of the GNN architecture

To assess the impact of the GNN architecture and especially of the convolutional layer part, we also trained different GNN using different convolutional layers than the one used in the original papers. More precisely, we trained the BreastCancer dataset with:

- graph convolutional layer (GCN) [22]
- GraphSage layer [35]

Again, we chose to test this variation on BreastCancer only for the sake of simplicity and because its relatively moderate size yields to reasonable computational times.

### Performance comparison

All scripts were run on a machine with a 1.90GHz Intel Xeon Bronze 3204 CPU with 12 cores and 128 Go of RAM. All (Python and R) CPU scripts were run sequentially (folds were processed one by one) to obtain the total computational time of the method and to avoid increasing the memory consumption artificially. The CPU scripts were all run on a single CPU core, except Random Forest and MLP (from scikit-learn). MLP is run by default using several CPU cores and the Random Forest method is indeed designed for parallel training as it is based on a large number of independent bootstrap samples. The Random Forest function from the R package randomForest is not natively made for parallel computation so we ran it sequentially (which explains the large difference in computational time between the two implementations; see Results).

For every simulation run, we kept the following:

- The computational (CPU) time for the training of the approach, the computational time to obtain predictions, the maximum (for Python scripts) and total (for R scripts) memory load. Python memory load was tracked using the Python module memory-profiler and R memory load using the R package profmem.
- For classification problems, the cross-validation accuracy, balanced accuracy and ROC AUC (using the one-versus-rest approach for multi-class problems).
- For regression problems, the cross-validation mean square error, squared correlations between predicted and true values, and explained variance.

## RESULTS

### Results on standard benchmarks from the literature

**Fig. 1:**
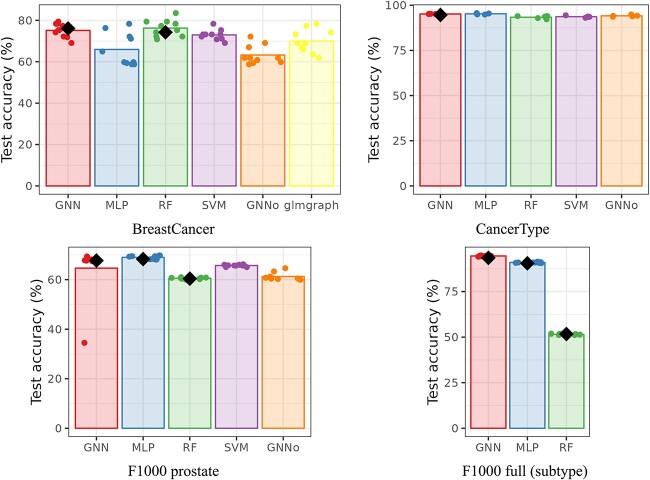
Cross-validation accuracy. The barplot height corresponds to the average accuracy across all folds, the dots to the individual accuracy of each fold and the black diamond (when present) to the averaged accuracy reported by the authors.

**Fig. 2:**
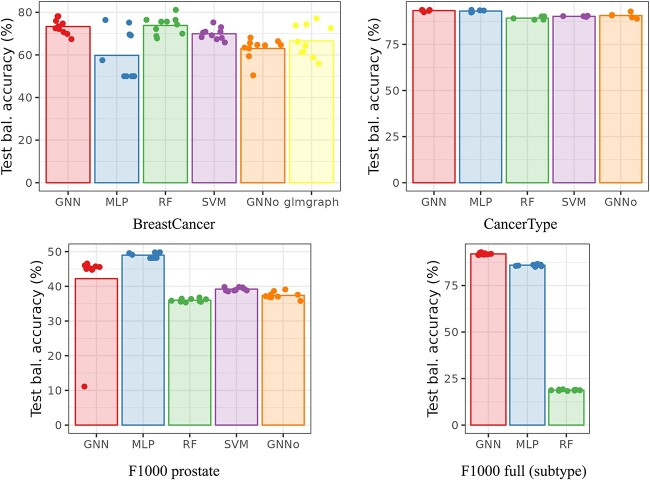
Cross-validation balanced accuracy. The barplot height corresponds to the average balanced accuracy across all folds and the dots to the individual balanced accuracy of each fold.

Figures 1 and 2, respectively, give the cross-validation accuracy (as reported in previous articles) and the cross-validation balanced accuracy (which accounts for differences in class frequencies) for all datasets across methods. In Figure 1, the average accuracy, as reported by the authors, is shown with a black diamond. BreastCancer results are those obtained on unscaled data ([8] reported similar results—slightly worse for AUC and slightly better for accuracy—on scaled data for their GNN). CancerType results are those obtained with the PPI network ([29] reported similar results for PPI+singleton network for their GNN), and F1000 results are those obtained for the prostate dataset (MOA prediction task), with the full graph and of the full dataset for the subtype prediction task (This is one of the prediction tasks for which GNN performed best in the original article. The results for the other tasks are provided in Section 3 of the Supplementary material.) Note that, for all reported results, we obtained performances very similar to the one obtained by the authors. Reproducibility of the published results and methods is thus good.

GNN repeatedly stands as a good method but, in all situations except for F1000 full, it is not the best from the accuracy point of view. Overall, considering that GNN is provided with the additional information of the relations between genes, this result can be considered as a poor one. This is especially true, since most of time the other methods (MLP, RF and SVM) were used *without* any hyperparameter tuning (default values provided by the method were used). Tuning of hyperparameters in GNN is often not described or commented and only final used hyperparameters are reported, which would tend to favor this approach. Note that this result is confirmed by the balanced accuracy, which can be considered as more faithful considering that the classes are not perfectly balanced. GNN shows a better balanced accuracy only for F1000 full and, by a very small margin compared to standard MLP, for CancerType. Finally, note that the poor result of RF in F1000 full is already commented in the original study of [9], who explained it by the higher heterogeneity of this dataset compared to the prostate specific dataset: random Forest might not be appropriate in this case, at least compared to MLP for instance.

In addition, over the different datasets, no clear winner method stands out and the difference of accuracy between the methods are very small in all cases. In particular, the CancerType performances are very stable across methods. This is explained by the fact that the prediction task (predicting the tissue of the cancer from gene expression) is an easy one (as is the prediction of the tissue from gene expression which are well separated by unsupervised methods as shown in [36]). These results are consistently observed whatever the quality criterion, the dataset preprocessing (scaled or not for BreastCancer), or the used network for CancerType (co-expression, PPI, with and without singletons), as shown in Section 3 of the Supplementary material. As for the other tasks of F1000 full (see Section 3.3 of the Supplementary material), similar conclusions can be drawn: RF behaves poorly in this case and this is the only situation in which GNN exhibits a small advantage over MLP, maybe explained by the higher variability of this dataset.

Finally, repeatedly, we found some outlier performance in some folds for GNN. When trying to investigate the problem more precisely, we found that it was only due to the random initialization of the training: changing the random seed leads to sometimes solving for this fold but frequently creating it for another fold. Folds with poor performances are systematically due to a training process stuck in a zone where the loss seems to be very flat and where the prediction function was constant. The same problem was observed for another implementation of ChebNet GNN, as shown in Section ‘Impact of the implementation and of the hyperpameters’ below.

**Fig. 3:**
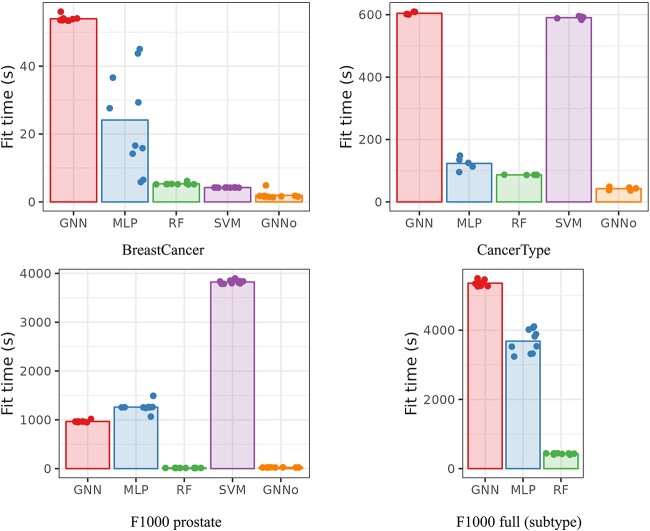
Computational time (in seconds) for training the different methods.

**Fig. 4:**
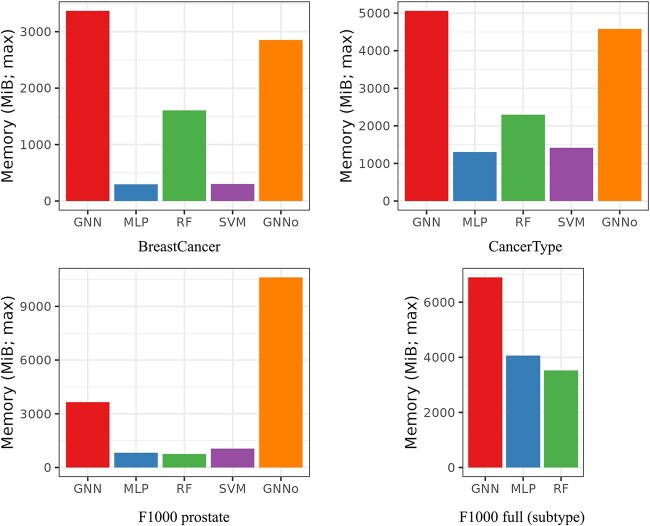
Maximum memory load during training (in MiB) for the different methods (Python implementation only).

Figures 3 and 4, respectively, give the computational times and the maximum memory load needed for training the different methods for each dataset. In Figure 3, the most computationally demanding method for BreastCancer (glmgraph, taking more than 30,000 s) is not represented for the sake of readability of the figure. In Figure 4, only methods implemented in Python (which excludes glmgraph) are represented to make sure that results are comparable but Supplementary material also provides the (total) memory load of R implementations computed with the R package profmem (Supplementary Section 3). Note that the two are not comparable because the Python module memory-profiler records the evolution of memory load over time whereas the R package profmem only records the total memory load of a given expression, which is much larger than the previous one.

As expected, SVM computational time is strongly influenced by the number of samples but not much by the number of genes. Note that, when the number of classes to predict is high (*e.g.* CancerType with 34 classes), the computational cost of SVM is also increased since a one-versus-one strategy is used for multiclass problems. GNN computational time is also often among the largest, except for the very small F1000 prostate dataset. Its computational time is more than twice that of MLP and seems to be strongly impacted by the number of nodes in the graph (see BreastCancer for which the number of nodes is 6888 but the sample size is small). RF and GNNo are constantly among the less demanding methods in terms of time. For the latter, it could seem surprising that the method is not impacted by the large number of samples in F1000 but it is fully explained by the number of hidden units of the model that we chose to keep to 100 whereas the selected “optimal” hyperparameter for MLP reported by the authors of [9] is of the order of ten times this value (see Tables S3 and S5 in Supplementary material).

From a memory load point of view, GNN and GNNo are the most demanding methods, GNNo being again strongly impacted by the number of samples. In contrast, SVM and MLP are not memory demanding and RF seems to rank in-between, providing an overall good trade-off between low computational time and low memory consumption for classification problems (results are a bit different for regression problems where deeper trees are usually built; see results on simulated datasets in Supplementary Sections 3.4 and 3.5).

Finally, Supplementary Section 3 also provides training accuracy, for which the conclusions are comparable to the test accuracy except for Random Forest: Random Forest is designed to interpolate data [37] and its training accuracy is thus equal to 100%.

### Impact of the implementation and of the hyperparameters

**Fig. 5:**
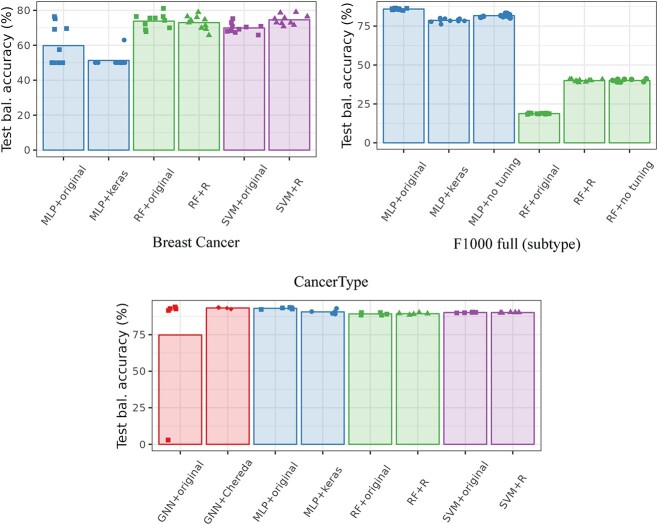
Cross-validation balanced accuracy for various implementations of the same method. The barplot height corresponds to the average balanced accuracy across all folds and the dots to the individual balanced accuracy of each fold.

Figure 5 gives the variation of cross-validated balanced accuracy across implementations for the different methods. In most situations, the achieved balanced accuracy was the same as with the original implementation, although some methods exhibited a variation depending on the implementation: MLP based on keras implementation was worse than the scikit-learn implementation for BreastCancer. On CancerType, the original implementation of the authors of [29] gave results in line with what was reported except for one fold which presents an unexplained very low performance. The implementation of [28] (called GNN+Chereda) on CancerType fixed the problem of the bad behavior of one of the folds and produced results comparable to MLP and to reported results. As previously discussed, we found that it was only due to the random initialization of the training.

Finally, on F1000 full for the subtype prediction task, the use of default parameters of the method with an increased number of trees provided a strong improvement of the accuracy, both for the R (RF+R in the figure) and for the scikit-learn (RF+no tuning in the figure) implementations (results are still not competitive with GNN or MLP for this dataset though). As expected, both SVM implementations (scikit-learn and R), based on the same C++ library, gave very similar results in all cases.

**Fig. 6:**
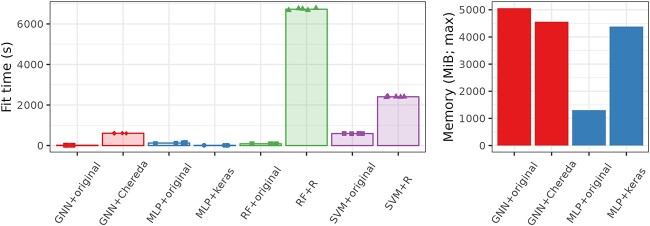
CancerType. Computational time (in seconds; left) and maximum memory load (in MiB; right) for training with various implementations.

However, these improvements came sometimes at the cost of a larger computational time or of a larger memory load as shown in Figure 6. In particular, in all cases, the R implementation was always much slower than the scikit-learn implementation (but this is partially explained by the fact that the scikit-learn implementation natively uses parallel computing). We also found that the memory load needed by the keras implementation of MLP is always much larger than the one needed by the scikit-learn implementation, which might be a side-effect of the fact that keras uses GPU computation and loads both CPU and GPU memory.

### Impact of network type

**Fig. 7:**
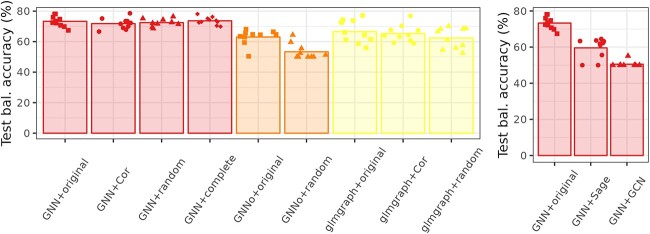
BreastCancer. Cross-validation balanced accuracy for graph-based methods with various input graphs and for GNN methods with varying architecture. The barplot height corresponds to the average balanced accuracy across all folds and the dots to the individual balanced accuracy of each fold.

The relatively low performance of GNN in the prediction task could be explained by a poor general behavior of the approach on this precise type of tasks or by poor inputs. More precisely, it can be explained by a poor accuracy of the gene interaction network. Figure 7 (left) illustrates the impact of irrelevant networks on the method balanced accuracy on BreastCancer. Note that **GNNo** and **glmgraph** could not be run with the complete network (between individuals) because they were both computationally too demanding.

For the two graph-based methods using gene networks (GNN and glmgraph), the impact of the input network is not visible. Surprisingly, for GNN, the complete network achieved better performance than networks based on biological knowledge (original, which is a PPI network) or on correlation between gene expression (which gave the worst results). glmgraph shows a slight improvement in using the PPI network but the difference remains small compared to the fold variations. For GNNo, using the true correlation network between individuals (patients) improves the performance compared to random network but these are still much lower than for the other two methods based on gene networks which means that using a patient network deteriorates the prediction performance compared to not using it (with Random Forest for instance). This confirms the poor usefulness of the input network, as already reported in the previous sections.

### Impact of the GNN architecture

Figure 7 (right) illustrates the type of GNN used on the prediction performance (balanced accuracy on BreastCancer). Compared with the original architecture proposed in [28], Sage or GCN convolution layers deteriorate the prediction performance. This is not a very surprising results because the published model is expected to have been carefully designed for the task and chosen among a variety of alternatives to achieve state-of-the-art results.

### Simulated and/or controlled data

**Fig. 8:**
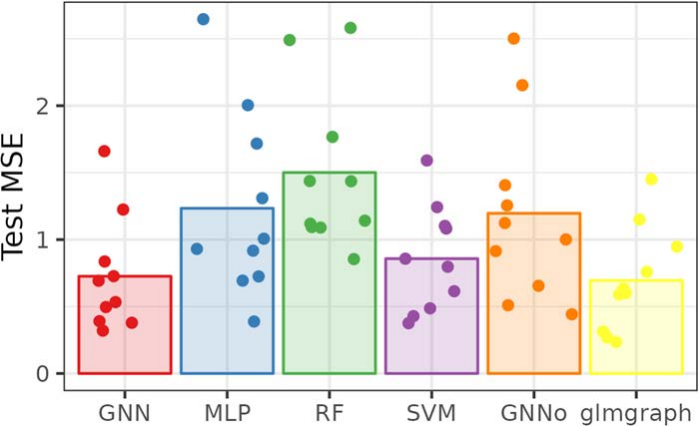
Simulated. Cross-validation mean squared error across methods.

**Fig. 9:**
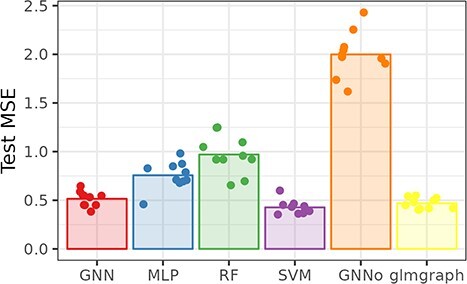
DREAM5. Cross-validation mean squared error across methods.

To better assess the impact of using a gene network for transcriptomic predictions, we relied on *in silico* expression data in which the network is indeed part of the expression generation process. Figures 8 and 9 display the mean square error (MSE) of the prediction task across methods, respectively for the Simulated and DREAM5 datasets.

Even if, probably due to the very small sample size, the MSE varies strongly, graph-based methods modeling relations between genes (GNN and glmgraph) indeed improve the prediction MSE over standard methods that do not use this information for the simulated dataset. This improvement is even clearer noticing that this prediction task can be considered as a very difficult one: Indeed, Supplementary Figure S28, which also reports cross-validated pseudo-$R^{2}$, shows negative average pseudo-$R^{2}$ for all methods except for GNN and glmgraph (and, to a lesser extent for SVM). Finally, GNNo, which only uses information of proximity between individuals does not provide a noticeable improvement compared to baseline ML methods (and also has an average negative $R^{2}$).

Similarly, graph predictions methods perform among the best for DREAM5, only competing with SVM for the best test MSE but much better, here, than MLP and RF. Again, GNNo has very low predictive performances.

**Fig. 10:**
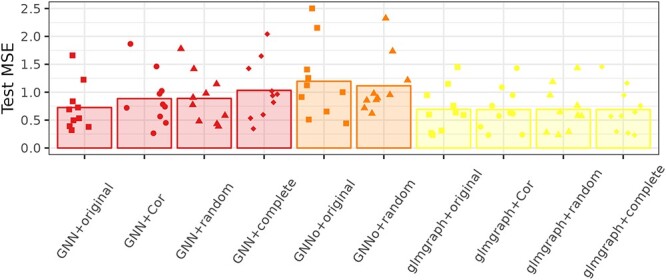
Simulated. Cross-validation mean squared error for graph-based methods with different input graphs.

Finally, Figure 10 shows the impact of using a non relevant network in the prediction for Simulated. For GNN, the impact of the complete network, which should be similar to the absence of additional information, is the worst, whereas the random graph has performance similar to the correlation network. This indicates that correlation networks, which is a method frequently used for network inference, probably provide information that is of low interest for the prediction purpose. Note that this impact is not visible on the glmgraph model, which is steadily providing the same MSE whatever the input network.

## DISCUSSION AND CONCLUSION

GNN has repeatedly been claimed to improve transcriptome-to-phenotype prediction by accounting for relations between genes, but our simulations show a slightly different story. Indeed, standard ML methods, not explicitly accounting for the dependency structure between genes, frequently obtain better or comparable performance on the prediction task. These results were found when comparing with both the GNN convolving on the gene network and the GNNo convolving on the sample network.

In addition, the computational efforts requested by the training of GNN are often overlooked: indeed, Table 4 compares the number of learned parameters for neural network-based methods and shows that the complexity of the trained GNN models is often very large. It is frequently of an order of magnitude larger than for the other approaches, which leads to an increased computational time and memory for learning.

**Table 4:** Total number of learned parameters for the neural network-based approaches (MLP, GNNo, GNN). The results are missing for GNNo for the F1000 full dataset due to memory issue as the graph between observations contains 156,461 nodes. OOM: out-of-memory.

**Total nb. params**		**MLP**	**MLP+keras**	**GNNo**	**GNN**
**BreastCancer**		689,102	689,502	1,378,302	41,166,018
**CancerType**	**PPI**	447,934	448,334	892,734	49,804,514 / 3,848,228 (Ramirez)
	**Spearman**	390,134	390,534	777,134	18,773,218 / 2,179,108 (Ramirez)
**F1000 prostate**	**MOA**	985,045	99,209	197,009	2,056,869
**F1000 full**	**MOA**	981,789	103,249	OOM	1,064,885
	**Subtype**	990,035	99,714	OOM	5,559,401
	**Primary Site**	988,039	99,512	OOM	4,751,043
**Simulated**		2301	271	481	49,697
**DREAM5**		156,601	157,001	313,401	19,866,689

The computational cost of the tuning of the model has never been included in the comparison: RF is known to be very insensitive to hyperparameter choices and default values for the few choices that have to be made (in combination with a reasonable—larger than 500—number of trees) is usually sufficient to obtain good performance for this method. On the contrary, the number of hyperparameters that have to be set for GNN is large: in addition to hyperparameters listed in Table S2 of Supplementary material, the architecture, type of convolution,... have to be designed. This design is rarely explained or documented but we can expect that it also comes at the cost of a large computational time and comparison with alternative architecture tends to show that the performance can be quite sensitive to these choices.

Our conclusion were not due to the fact that we were not able to reproduce GNN performances published by the authors. Most of the time, the provided implementation, description of hyperparameters and preprocessing were sufficient to recover the performances of GNN as published in the articles. However, the corresponding code and hyperparameters for concurrent ML methods were rarely given (with the exception of [9]) and not even performing hyperparameter tuning, we found that RF and MLP could obtain comparable or better performances. More surprisingly, using GNN in combination with irrelevant networks (random or complete) also led to comparable or better results than using the gold-standard network used by the authors (a fact that is already mentioned by [8]): This, itself, is enough to indicate that the graph information is not relevant for phenotype prediction in this model. Hence, in addition to speak in favor of a more complete report of the benefit of models by accounting for complexity and for the global computational cost evaluation, we want to point that reproducibility of some articles could be improved by a more complete report of the results: the complete code of the comparison should be provided (not just the code of the method) together with used libraries/functions/languages, hyperparameter values, fold definition (if CV is performed, which is desirable) and control of the randomness. The variability over folds is frequently reported but should also be discussed in comparison with the difference in average performances. In our experiments, the confidence interval of the average performance (as given by the folds) are frequently overlapping, showing no significant differences between method performances. In this situation, even a modification of the random seed for method incorporating some random process (as Random Forest) or a modification of the fold definition could change the ranking of the methods in term of performances.

CancerType shows surprisingly stable (and very good) results, over method choices but also hyperparameter variations for all methods (we even varied GNN hyperparameters for this dataset, with no variation of the performance in this case also). Thus, we think that this benchmark, which is frequently used to test classification methods from gene expression data, is probably not really appropriate for comparison purpose. Indeed, the first use of this dataset is from [38], who used it to propose a feature selection method that extracted biomarkers specific of the different cancer types. Hence, there is little practical interest in discussion classification performance on this dataset since i) the question of being able to predict the tissue (‘type’) of a given tumor from expression data has low (if any) medical interest (this tissue is usually known by design) and ii) the performance of a classification method is not directly related to the relevance of biomarkers that can be extracted from these methods (typically, methods including feature selection in the model design, like the one described in [38], are expected to show lower classification accuracy that methods using all gene expressions like the GNN approach discussed in [29]). Very few datasets are readily available to benchmark the usefulness of PPI or regulatory networks in expression-to-phenotype prediction tasks: the present work relies on three published datasets and two simulated dataset and none of them was based on the promising single-cell transcriptomic technology. Providing the community with a larger and more diverse benchmark, augmented with other datasets than the ones we have provided in this article, and/or organizing a challenge (as DREAM) would be valuable to fairly discuss the development of methods like GNN.

Finally, our simulated dataset examples gave some hope for methods using gene networks for phenotype prediction: in the smallest example, the network used for the training is the one which actually generated our synthetic phenotype. When this ground true network is used in graph-based models, it indeed improves the performance. Even if GNN do not outperform SVM and glmgraph in DREAM5, it shows much larger improvement over MLP and RF than in real-life datasets. Its lower performance for DREAM5 (compared to Simulated) could be explained by the larger dataset size, which increases the number of convolution parameters of these models, or to a prediction task less directly related to the network: in Simulated, the target is a protein level at time $t$ generated from gene expression at time $t-1$, whereas DREAM5 is a steady-state problem in which the target and gene expression are observed at the same time step. Since expression regulation is a dynamic process, this could reduce the network impact in the prediction.

Moreover, a recent benchmark [39] obtained competitive performance for the prediction of ncRNA-disease associations with GNN but this problem is formulated in a different way where the GNN task is to predict links in the input graph and not an external value using the graph: it might be worth of interest to investigate if, more generally, GNN might be more adapted to link prediction tasks than to graph property prediction tasks.

**Key Points**

- Graph neural network (GNN) has repeatedly been claimed to improve transcriptomic prediction by accounting for relations among genes.
- However, benchmarking with real datasets shows similar performances for simpler machine learning methods than for GNN.
- In addition, benchmarking with real expression datasets and irrelevant networks do not show decrease in performance compared to using a biologically relevant gene network.
- Benchmarking with simulated data shows that GNN might be relevant when the gene network is perfectly known.
- The lack of improvement for GNN with real data might be due to the low accuracy of available gene networks.

## Supplementary Material

brouard_etal_p2022-suppmat_bbae027

## Data Availability

The data underlying this article were provided as described articles [8, 9, 29] (see details in Table 2) or, if produced by authors of the present article, they are available in recherche.data.gouv.fr at https://doi.org/10.57745/BZ0TTC. The scripts underlying this article are available at https://forgemia.inra.fr/nathalie.villa-vialaneix/gnn.git.

## Author Biographies

**Céline Brouard** is a permanent researcher at INRAE and develops machine learning approaches for bioinformatics.

**Raphaël Mourad** is an associate professor at University Paul Sabatier working on machine learning for genomics.

**Nathalie Vialaneix** is a senior researcher (directrice de recherche) at INRAE. She develops statistics and machine learning methods for omics data analysis and integration.
