# Supplementary material for: Should we really use graph neural networks for transcriptomic prediction?
Source: Brief Bioinform. 2024 Feb 12;25(2):bbae027. doi: 10.1093/bib/bbae027 (PMC10939369; doi:10.1093/bib/bbae027)
Supplement: brouard_etal_p2022-suppmat_bbae027 [file brouard_etal_p2022-suppmat_bbae027.pdf]

Supplementary information for “Should we really use graph neural networks for transcriptomic prediction?”

Céline Brouard, Raphaël Mourad, and Nathalie Vialaneix

Contents

1 Datasets **2**
1.1 Description . . . 2
1.2 Description of preprocessing performed on the different datasets . . . 2
1.2.1 BreastCancer . . . 2
1.2.2 CancerType . . . 2
1.2.3 F1000 . . . 2
1.2.4 Simulated . . . 2
1.2.5 DREAM5 . . . 3

2 Hyperparameters **3**

3 Detailed results **8**
3.1 BreastCancer . . . 8
3.1.1 Unscaled data . . . 8
3.1.2 Implementations (unscaled data) . . . 9
3.1.3 Graphs (unscaled data) . . . 10
3.1.4 GNN type (unscaled data) . . . 11
3.1.5 Computational efficiency (time and memory) . . . 12
3.1.6 Effect of implementation on computational efficiency . . . 13
3.1.7 Effect of the GNN type on computational efficiency . . . 14
3.1.8 Effect of the graph on computational efficiency . . . 15
3.1.9 Scaled data . . . 16
3.2 CancerType . . . 17
3.2.1 PPI + singleton . . . 17
3.2.2 Implementation PPI + singleton . . . 18
3.2.3 Computational efficiency (time and memory) . . . 19
3.2.4 PPI . . . 20
3.2.5 Spearman . . . 21
3.2.6 Implementation Spearman . . . 22
3.3 F1000 . . . 23
3.3.1 Prostate . . . 23
3.3.2 Implementation (prostate) . . . 24
3.3.3 Computational efficiency (time and memory) for prostate . . . 25
3.3.4 Full + subtype . . . 26
3.3.5 Implementation (full + subtype) . . . 27
3.3.6 Computational efficiency (time and memory) for full + subtype . . . 28
3.3.7 Full + primary site . . . 29
3.3.8 Implementation (full + primary site) . . . 30
3.3.9 Computational efficiency (time and memory) for full + primary site . . . 31
3.3.10 Full + MOA . . . 32
3.3.11 Implementation (full + MOA) . . . 33
3.3.12 Computational efficiency (time and memory) for full + MOA . . . 34
3.4 Simulated (sismonr) . . . 35
3.4.1 Methods comparison . . . 35
3.4.2 Implementation . . . 36
3.4.3 Graphs . . . 37
3.4.4 Computational efficiency (time and memory) . . . 38
3.4.5 Effect of implementation and of the graph on computational efficiency . . . 39

3.5 Simulated (DREAM5, scaled data) 40
3.5.1 Methods comparison 40
3.5.2 Implementation 41
3.5.3 Computational efficiency (time and memory) 42

1 Datasets

1.1 Description

Table S1: Description of datasets.

Dataset	Gene network	Type of transcriptomic data	Prediction goal
**BreastCancer**	PPI network (HPRD)	microarray	Prediction of metastasis within the first five years in breast cancer.
**CancerType**	PPI network (STRING) and co-expression network based on Spearman correlation	RNA-seq data (TCGA)	Classification of different tumor and non-tumor samples into 33 cancer "types" (actually tissue in which the tumor has been found) or as normal.
**F1000**	Regulatory network (RegNetwork)	L1000 assay (measurement of a reduced representation of the transcriptome) on cell lines, profiled in different tissues and with different drugs	Prediction of primary site (tissue type), subtype (e.g., "malignant melanoma" or "myoblast", related to a disease state) and drug mechanism of action.
**Simulated**	Given network	Simulated data (obtained from the network and simulating from mRNA / protein quantities dynamic relations) by **sismonr**	Prediction of one protein quantity at time t from mRNA quantities at time $t-1$.
**DREAM5**	Given network	Simulated static expression data (obtained from the network)	Prediction of one gene expression based on the expression of the other genes.

1.2 Description of preprocessing performed on the different datasets

1.2.1 BreastCancer

In [1], the authors reported results based on "standardized" and "non standardized" data for GNN and RF (results of [2] seem to have been obtained from standardized data). If $(\mathbf{X}_{ij})_{i=1,\ldots,n,\ j=1,\ldots,p}$ is the gene expression matrix for observation (patient) i and variable (gene) j, "non standardized" data correspond to substracting the minimum expression from $\mathbf{X}$:

$$\forall\, i,\, j,\ \tilde{\mathbf{X}}_{ij} := \mathbf{X}_{ij} - m, \qquad \text{where } m = \min_{ij} \mathbf{X}_{ij},$$

such that all expressions lie in $[0, 8.35]$.

"Standardized data" correspond to a centering and scaling of the original data:

$$\forall\, i,\, j,\ \tilde{\tilde{\mathbf{X}}}_{ij} := \frac{\mathbf{X}_{ij} - \overline{\mathbf{X}}_j}{\sigma_j}, \quad \text{where } \overline{\mathbf{X}}_j = \frac{1}{n}\sum_{i=1}^{n} \mathbf{X}_{ij} \text{ and } \sigma_j^2 = \frac{1}{n}\sum_{i=1}^{n}\left(\mathbf{X}_{ij} - \overline{\mathbf{X}}_j\right)^2. \tag{1}$$

Note that the random forest should give identical results using either $\tilde{\mathbf{X}}$ or $\tilde{\tilde{\mathbf{X}}}$ since both are linear transformations of the original variables and don't change the definition (variable and threshold) of the optimal split. Hence, differences in performance reported between the two datasets in [1] is probably just the effect of the randomness of the method (when an identical random seed has not been set for the training of both datasets).

1.2.2 CancerType

No further preprocessing of the data (compared to available dataset), either in [3] or in our experiments.

1.2.3 F1000

No further preprocessing of the data (compared to dataset sent by authors) in our experiments.

1.2.4 Simulated

As compared with the output of the **sismonr** package, inputs and outputs were centered and scaled to unit variance as described in Equation (1).

1.2.5 DREAM5

As compared with original dataset, inputs and outputs were centered and scaled to unit variance as described in Equation (1).

2 Hyperparameters

**Hyperparameter**	**BreastCancer**	**CancerType**	**F1000 prostate MOA**	**F1000 full**			**Simulated**	**DREAM5**
				**MOA**	**Subtype**	**Primary Site**		
Number of epochs	100	20	200	350	300	350	100	100
Batch size	109	200	55	92	88	68	100	109
Learning rate	0.001	0.001/0.005	0.005	$1.23\ 10^{-3}$	$2.95\ 10^{-3}$	$3.13\ 10^{-3}$	0.001	0.001
Decay rate	0.95	0.95	0.95	0.991	0.976	0.989	0.95	0.95
Decay steps	$\frac{n\_train}{batch\_size}$	17.7	415	405	362	380	$\frac{n\_train}{batch\_size}$	$\frac{n\_train}{batch\_size}$
Dropout	1	1	0.5	0.698	0.454	0.562	1	1
Regularization parameter	10^{-4}	0	$4.0\ 10^{-3}$	$1.09\ 10^{-2}$	$5.42\ 10^{-3}$	$3.08\ 10^{-3}$	10^{-4}	10^{-4}
Momentum	no	no	0.97	0.879	0.973	0.945	no	no
Number of GC layers	2	1	1	1	1	1	1	1
Number of GC filters	[32, 32]	1	25	9	43	41	32	[32, 32]
Polynomial order	[8, 8]	1	15	7	8	5	8	[8, 8]
Pooling sizes	[2, 2]	2	2	2	2	2	2	[2, 2]
Pooling type	max	averaged	max	averaged	max	averaged	max	max
Size of dense layers	[512, 128]	1024	[168, 14]	137	[150, 150]	135	128	[512, 128]

Table S2: **GNN**: Chosen hyper-parameters for the different datasets. When the number of graph convolutional (GC) or dense layers is greater than one, brackets are used to indicate the value of the hyperparameter for each layer.

**Hyperparameter**	**BreastCancer**	**CancerType**	**F1000 prostate MOA**	**F1000 full**			**Simulated**	**DREAM5**
				**MOA**	**Subtype**	**Primary Site**		
Number of epochs	100	100	100	100	100	100	100	100
Batch size	200	200	200	200	200	200	32	200
Learning rate (Adam)	0.001	0.001	0.001	0.001	0.001	0.001	0.001	0.001
Decay rate beta_1 (Adam)	0.9	0.9	0.9	0.9	0.9	0.9	0.9	0.9
Decay rate beta_2 (Adam)	0.999	0.999	0.999	0.999	0.999	0.999	0.999	0.999
Epsilon (Adam)	10^{-8}	10^{-8}	10^{-8}	10^{-8}	10^{-8}	10^{-8}	10^{-8}	10^{-8}
Dropout	0	0	0	0	0	0	0	0
Regularization parameter	10^{-4}	10^{-4}	10^{-4}	10^{-4}	10^{-4}	10^{-4}	10^{-4}	10^{-4}
Number of GC layers	1	1	1	1	1	1	1	1
Number of GC filters	100	100	100	100	100	100	10	100

Table S3: **GNNo**: Chosen hyper-parameters for the different datasets.

Hyperparameter	BreastCancer	CancerType	F1000 prostate MOA	F1000 full MOA	F1000 full Subtype	F1000 full Primary Site	Simulated	DREAM5
Perceptron								
Used function	MLPClassifier						MLPRegressor	
	(scikit-learn)							
Random forest								
Used function	RandomForestClassifier						RandomForestRegressor	
	(scikit-learn)							
n_estimators	500							
bootstrap	False							
Random forest (R)								
Used function	randomForest							
replace	FALSE							
sampsize	length(training)							
nodesize	default value						1	
SVM								
Used function	SVC						SVR	
	(scikit-learn)							
SVM (R)								
Used function	svm							
	(e1071)							
type	C-classification						eps-regression	
gamma	1/(ncol(training)*var(training))							
glmgraph								
standardize	FALSE	NC					FALSE	
family	binomial	NC					gaussian	
type.measure	auc	NC					mse	

Table S4: **Perceptron**, **Random forest**, **SVM**, and **glmgraph** (whenever relevant): Chosen hyper-parameters for the different datasets. Unspecified hyper-parameters were set to their default values.

**Hyperparameter**	**F1000 prostate MOA**	**F1000 full MOA**	**F1000 full Subtype**	**F1000 full Primary Site**
Perceptron				
Used function	`MLPClassifier` (scikit-learn)	`MLPClassifier` (scikit-learn)		
learning_rate	invscaling	adaptive	invscaling	invscaling
hidden_layer_sizes	[997]	[955]	[997]	[997]
learning_rate_init	0.0553037462469582	0.10898026569061127	0.0553037462469582	0.0553037462469582
momentum	0.8670995345995152	0.86370430284520194	0.8670995345995152	0.8670995345995152
alpha	0.8200640684863673	1.6879174708893805	0.8200640684863673	0.8200640684863673
power_t	0.2260141186252257	0.33021989249044037	0.2260141186252257	0.2260141186252257
beta_1	0.864394587085052	0.919473939313036	0.864394587085052	0.864394587085052
beta_2	0.9985546737363457	0.99919431656457547	0.9985546737363457	0.9985546737363457
max_iter	350	164		
epsilon	8.57966387882825e-09	9.7045902021921481e-10	8.57966387882825e-09	8.57966387882825e-09
solver	sgd	sgd	sgd	sgd
early_stopping		True		
Random forest				
Used function	`RandomForestClassifier` (scikit-learn)	`RandomForestClassifier` (scikit-learn)		
criterion	entropy			entropy
min_weight_fraction_leaf	6.00547769973228e-05	1.2722643563513202e-06	0.00043290898297300624	0.0003865563609192391
min_samples_leaf	2			
min_impurity_decrease	0.0003681428790609335	1.6979071770883573e-05	3.637973058798681e-05	0.00028912503549492427
n_estimators	53	211	411	401
max_depth		100	25	100
max_leaf_nodes			500	
min_samples_splits				4

Table S5: **Perceptron** and **Random forest**: Hyper-parameters as set in the original article [4] (empty fields indicate that the default value has been used).

3 Detailed results

3.1 BreastCancer

3.1.1 Unscaled data

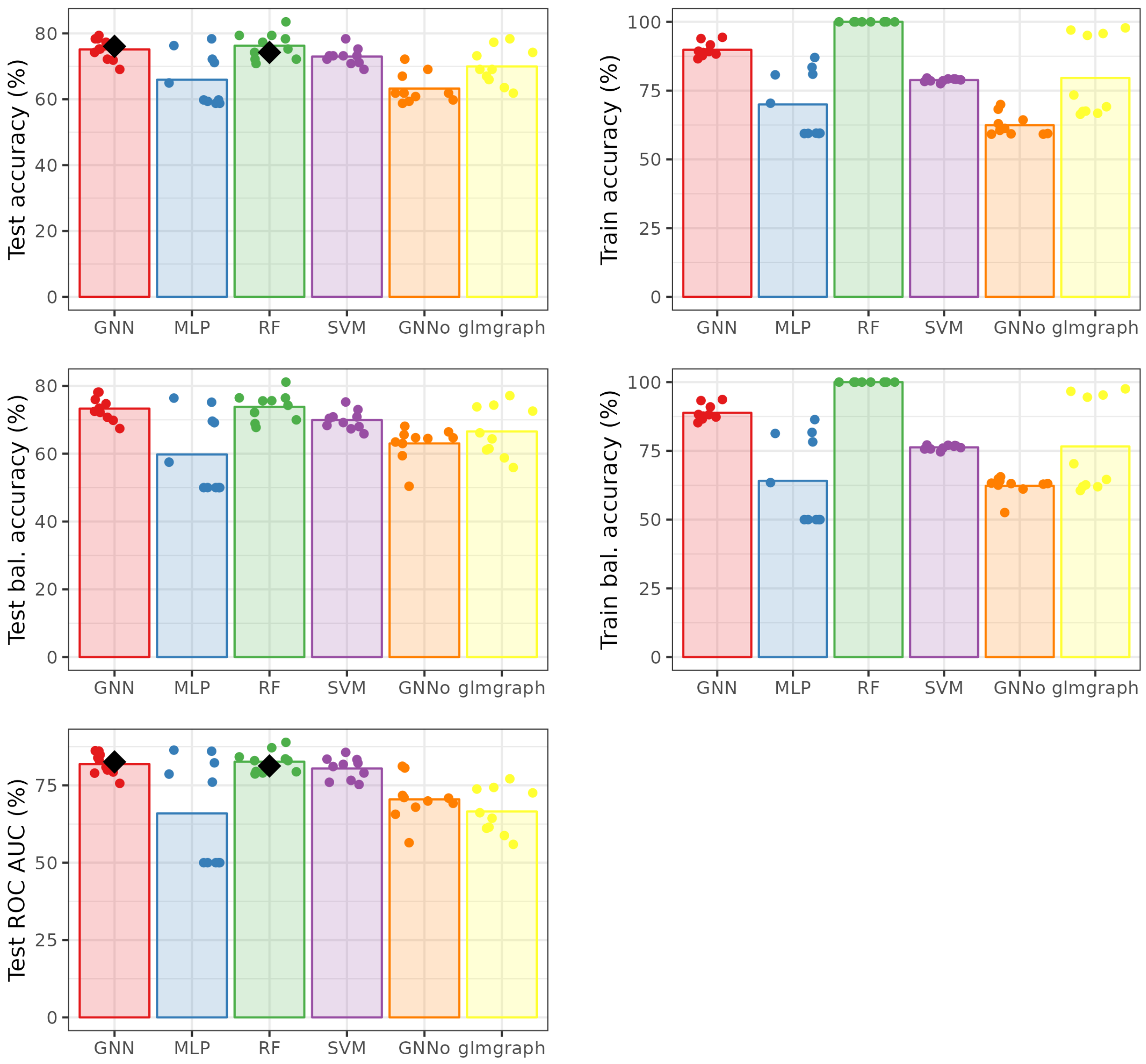

Figure S1: **BreastCancer.** Unscaled data: CV (left) and train (right) accuracy, balanced accuracy, and AUC ROC.

3.1.2 Implementations (unscaled data)

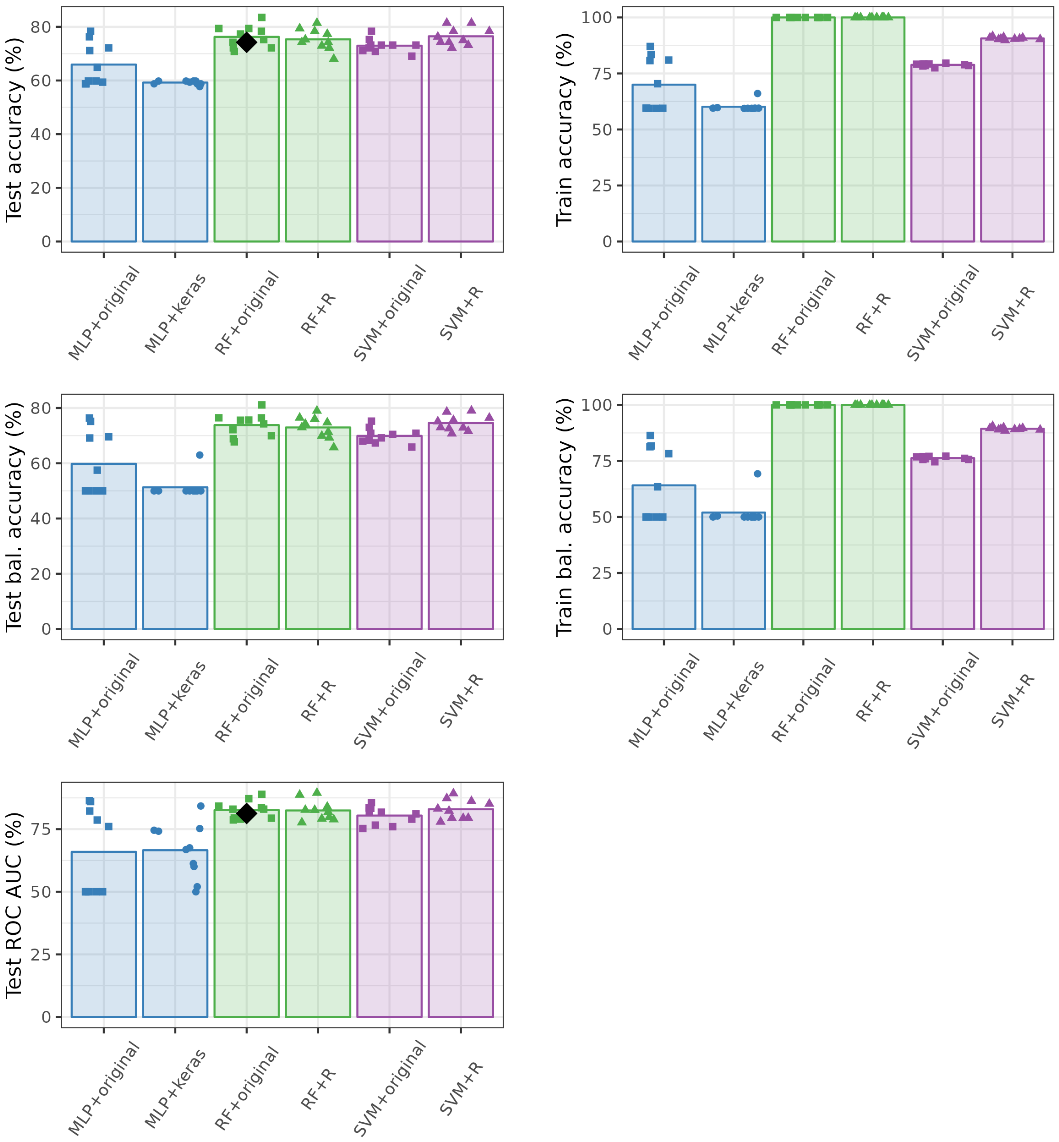

Figure S2: **BreastCancer.** Unscaled data: CV (left) and train (right) accuracy, balanced accuracy, and AUC ROC for varying implementations.

3.1.3 Graphs (unscaled data)

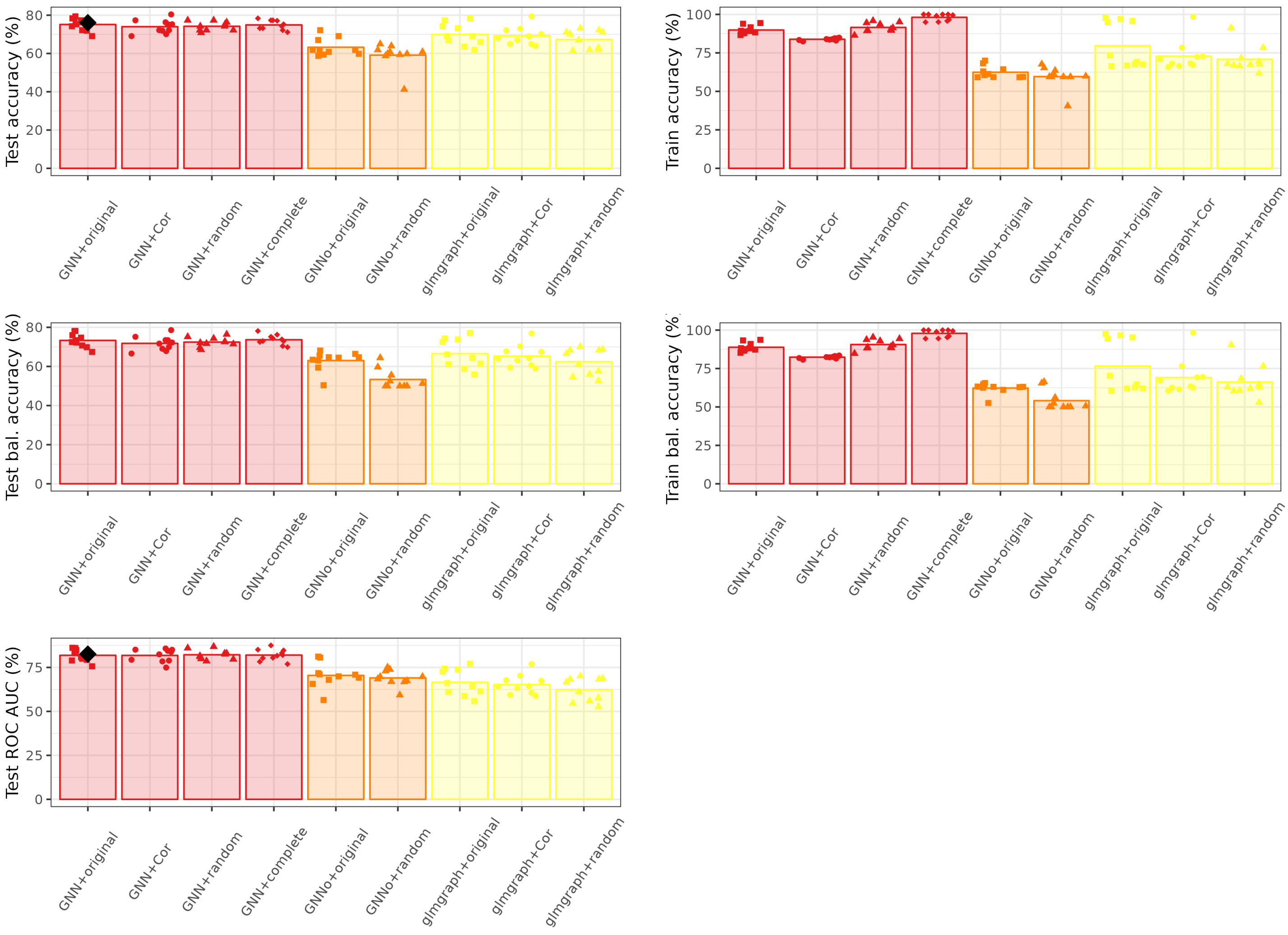

Figure S3: **BreastCancer.** Unscaled data: CV (left) and train (right) accuracy, balanced accuracy, and AUC ROC for varying input graphs.

3.1.4 GNN type (unscaled data)

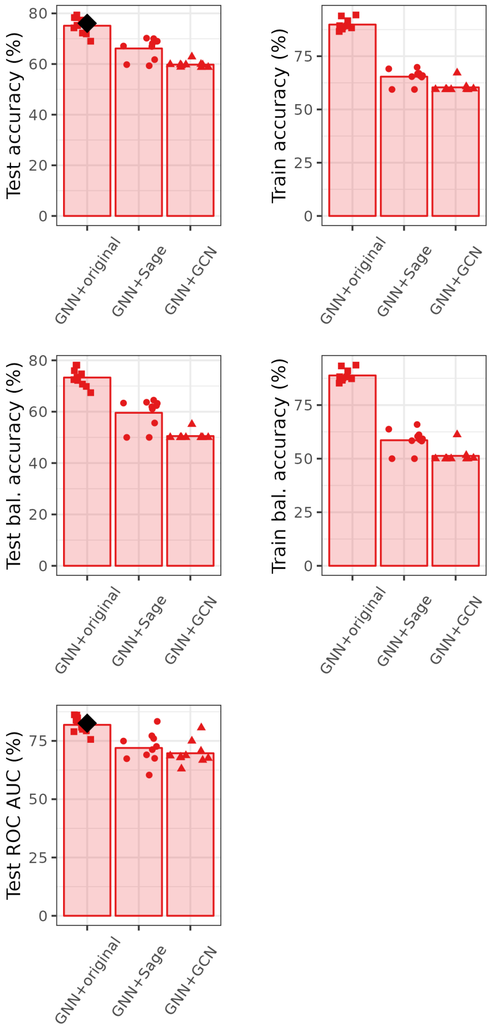

Figure S4: **BreastCancer.** Unscaled data: CV (left) and train (right) accuracy, balanced accuracy, and AUC ROC for varying convolution layers.

3.1.5 Computational efficiency (time and memory)

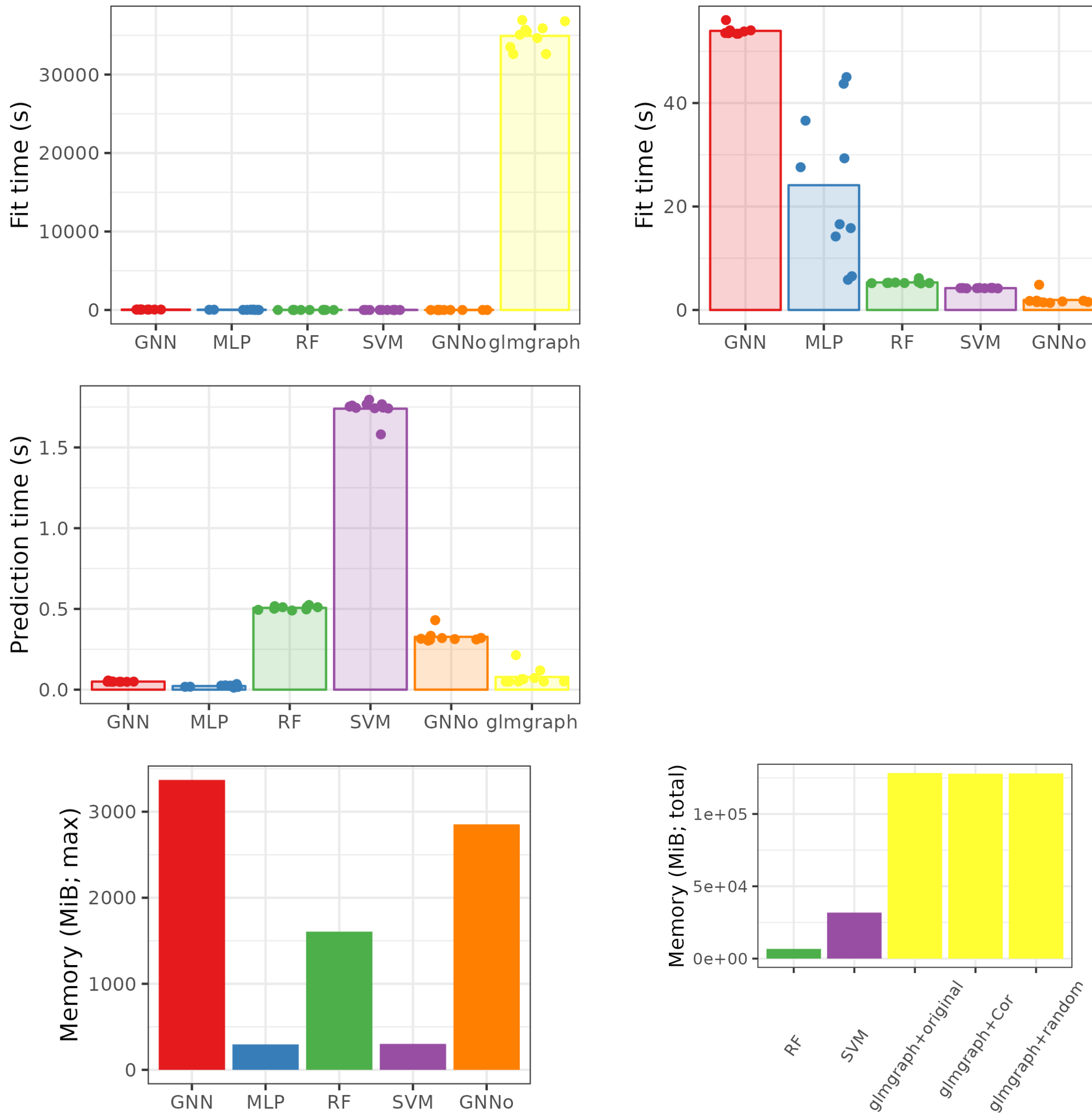

Figure S5: **BreastCancer.** Unscaled data: Fit time with and without glmgraph (first row), prediction time (second row), and maximum or total memory load (in MiB), respectively for Python scripts (left) and R scripts (right), for varying input graphs.

3.1.6 Effect of implementation on computational efficiency

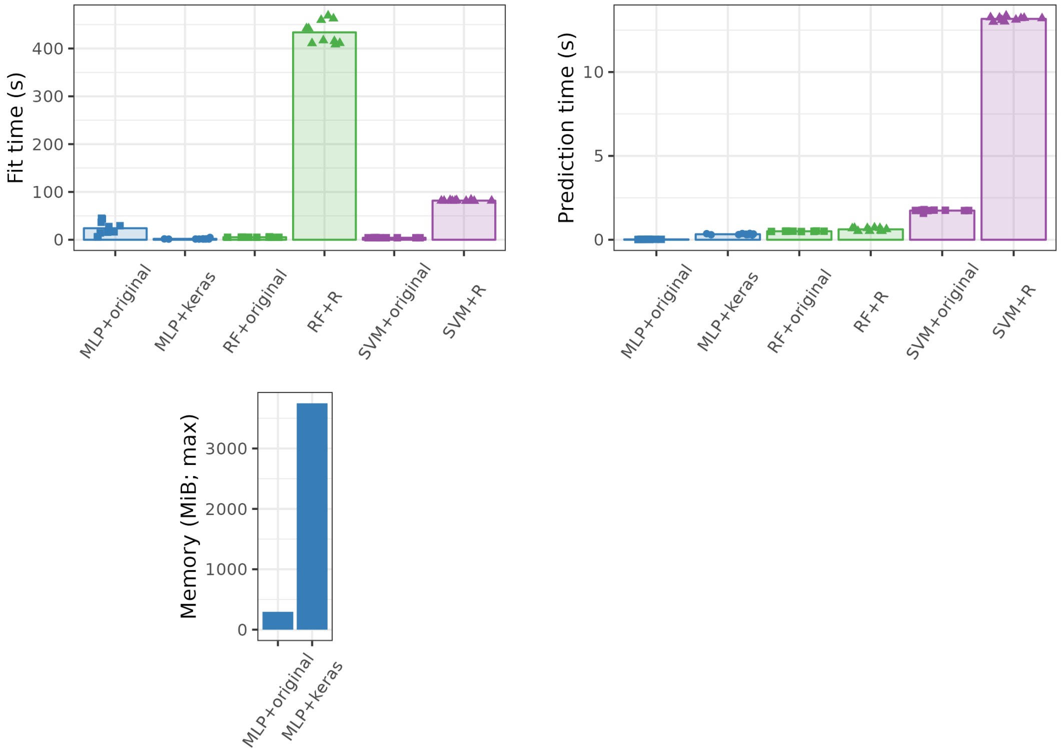

Figure S6: **BreastCancer.** Unscaled data: Fit and prediction times (first row), and maximum memory load (in MiB) for Python scripts (second row) for varying implementations.

3.1.7 Effect of the GNN type on computational efficiency

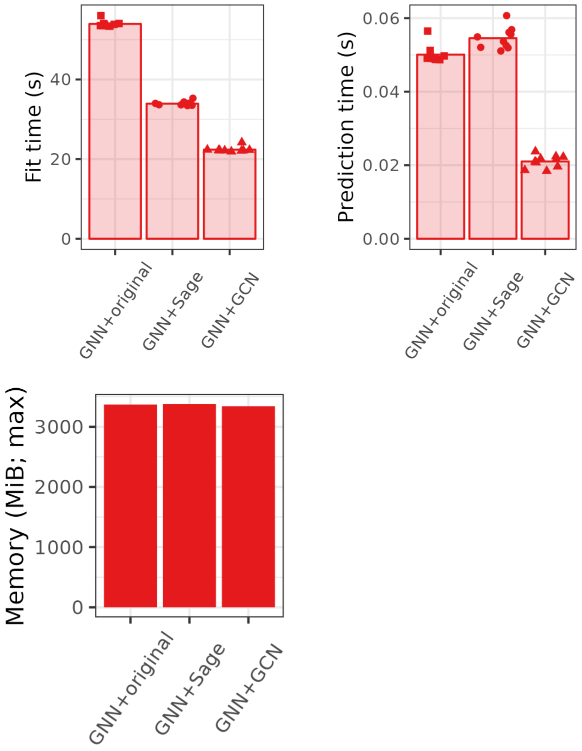

Figure S7: **BreastCancer.** Unscaled data: Fit and prediction times (first row), and maximum memory load (in MiB) for Python scripts (second row) for varying convolutional layers.

3.1.8 Effect of the graph on computational efficiency

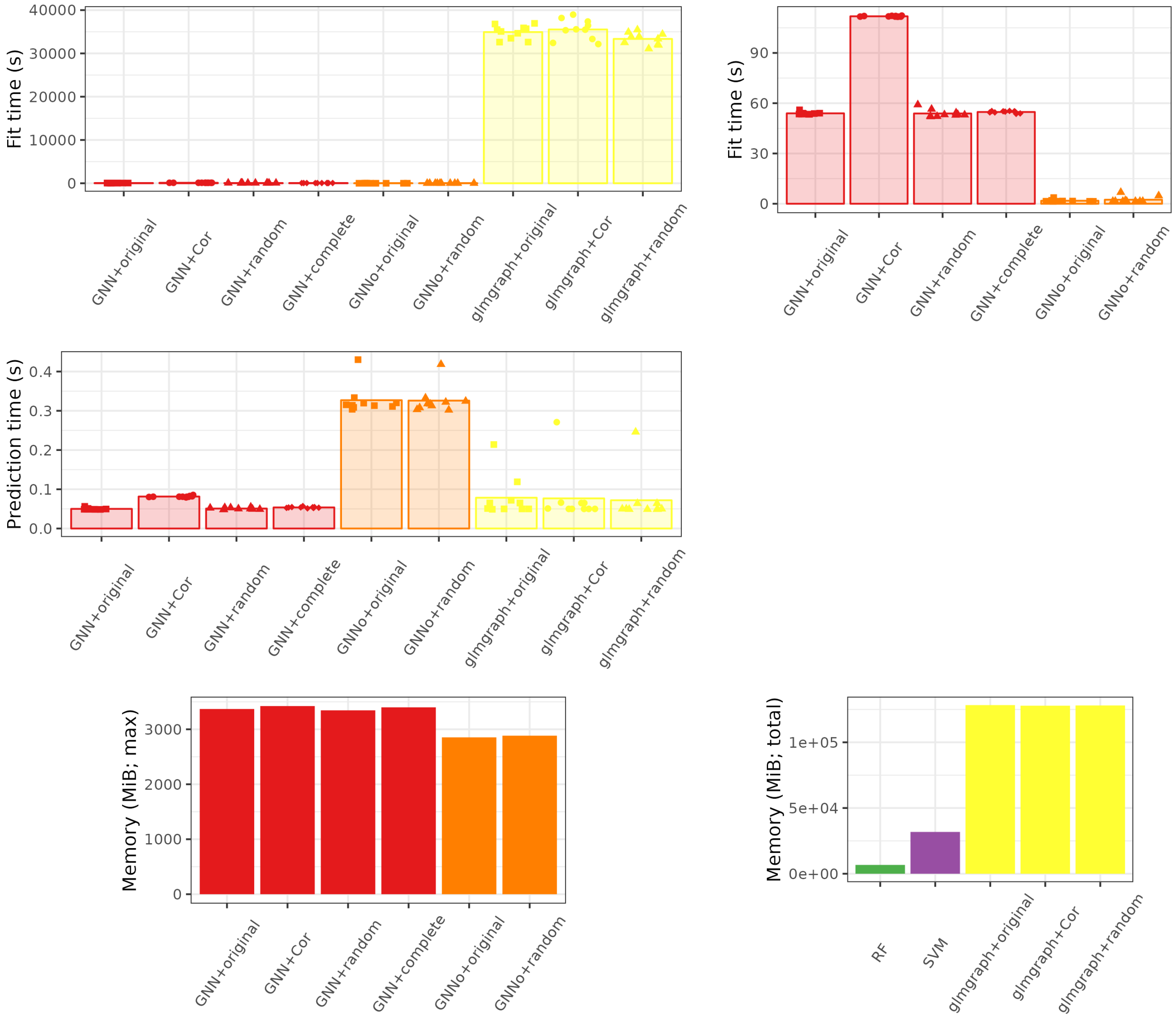

Figure S8: **BreastCancer.** Unscaled data: Fit time with and without glmgraph (first row), prediction time (second row), and maximum or total memory load (in MiB), respectively for Python scripts (left) and R scripts (right).

3.1.9 Scaled data

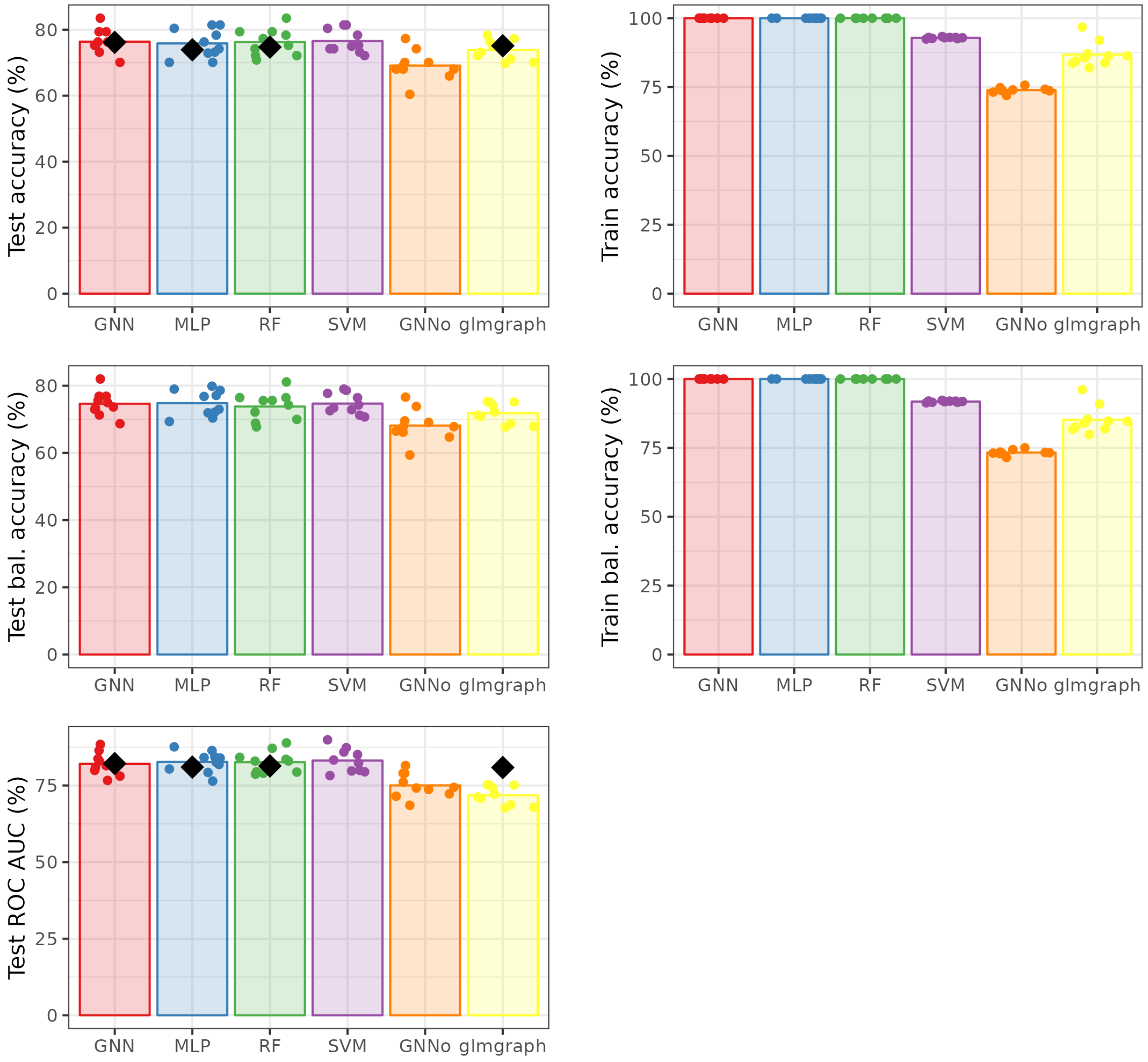

Figure S9: **BreastCancer.** Scaled data: CV (left) and train (right) accuracy, balanced accuracy, and AUC ROC.

3.2 CancerType

3.2.1 PPI + singleton

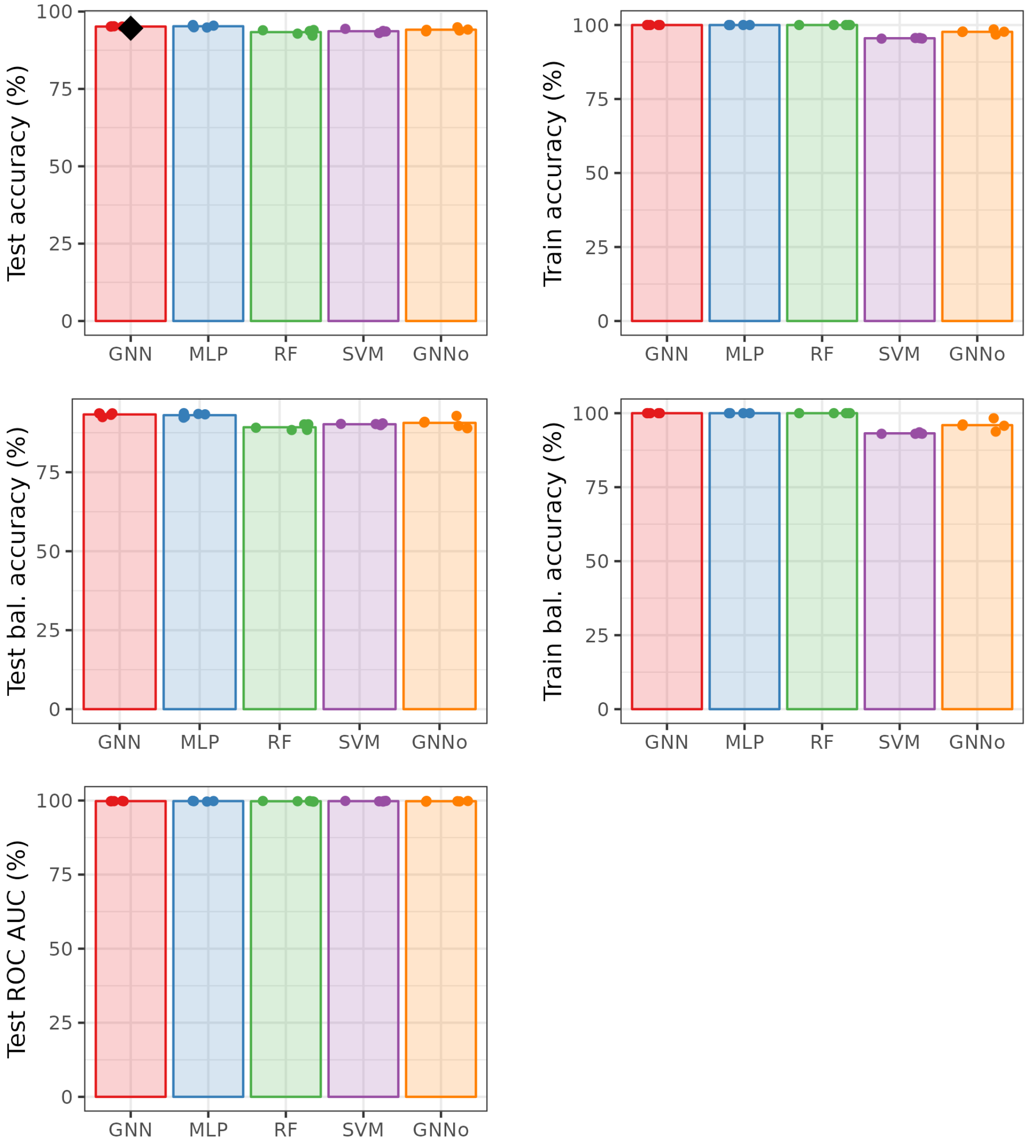

Figure S10: **CancerType.** PPI+singleton data: CV (left) and train (right) accuracy, balanced accuracy, and AUC ROC.

3.2.2 Implementation PPI + singleton

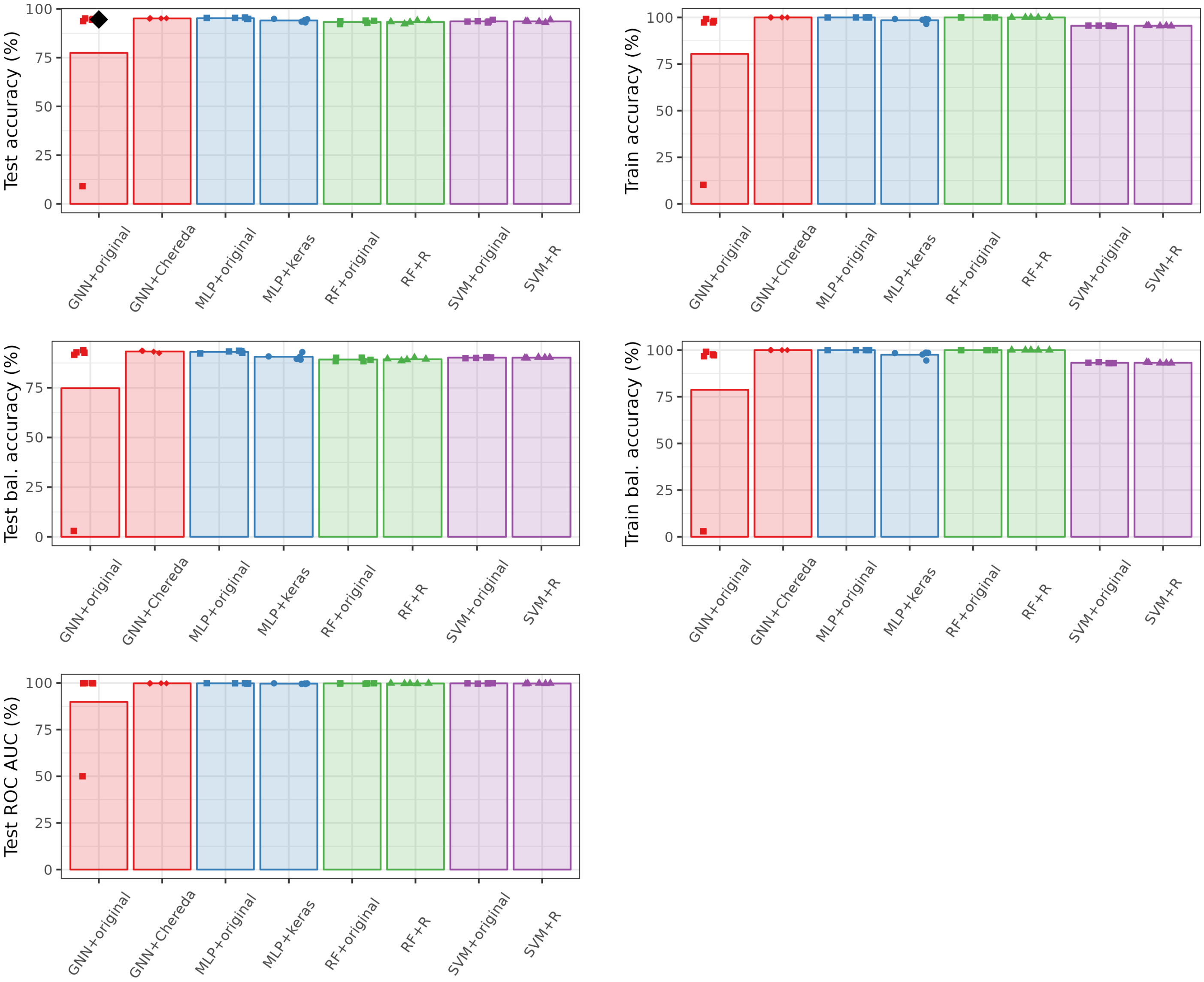

Figure S11: **CancerType.** PPI+singleton data: CV (left) and train (right) accuracy, balanced accuracy, and AUC ROC for varying implementations.

3.2.3 Computational efficiency (time and memory)

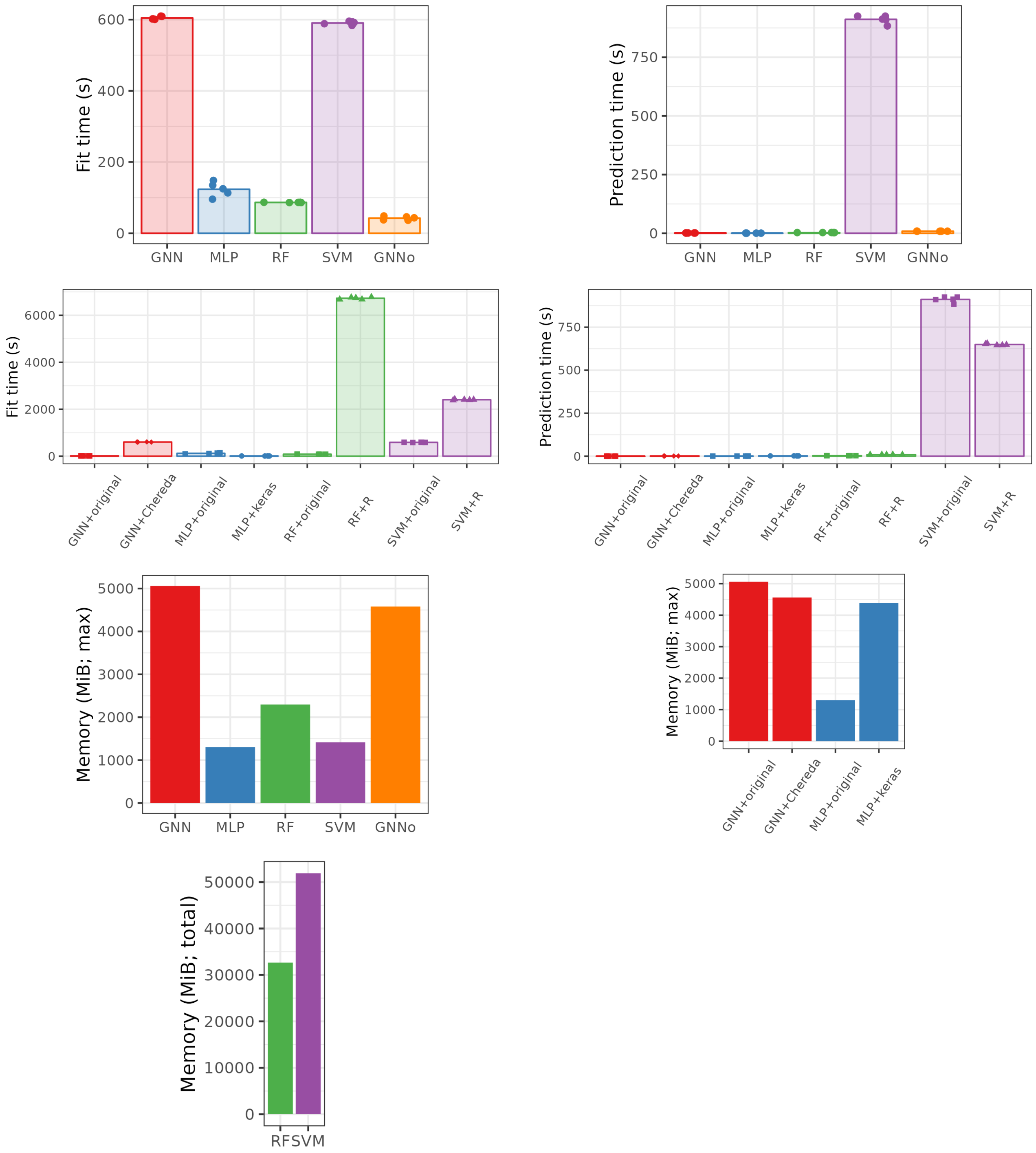

Figure S12: **CancerType.** PPI+singleton data: Fit and prediction times (first row), effect of the implementation on fit and prediction times (second row), maximum memory load (in MiB) for Python scripts (third row), and total memory load (in MiB) for R scripts (fourth row).

3.2.4 PPI

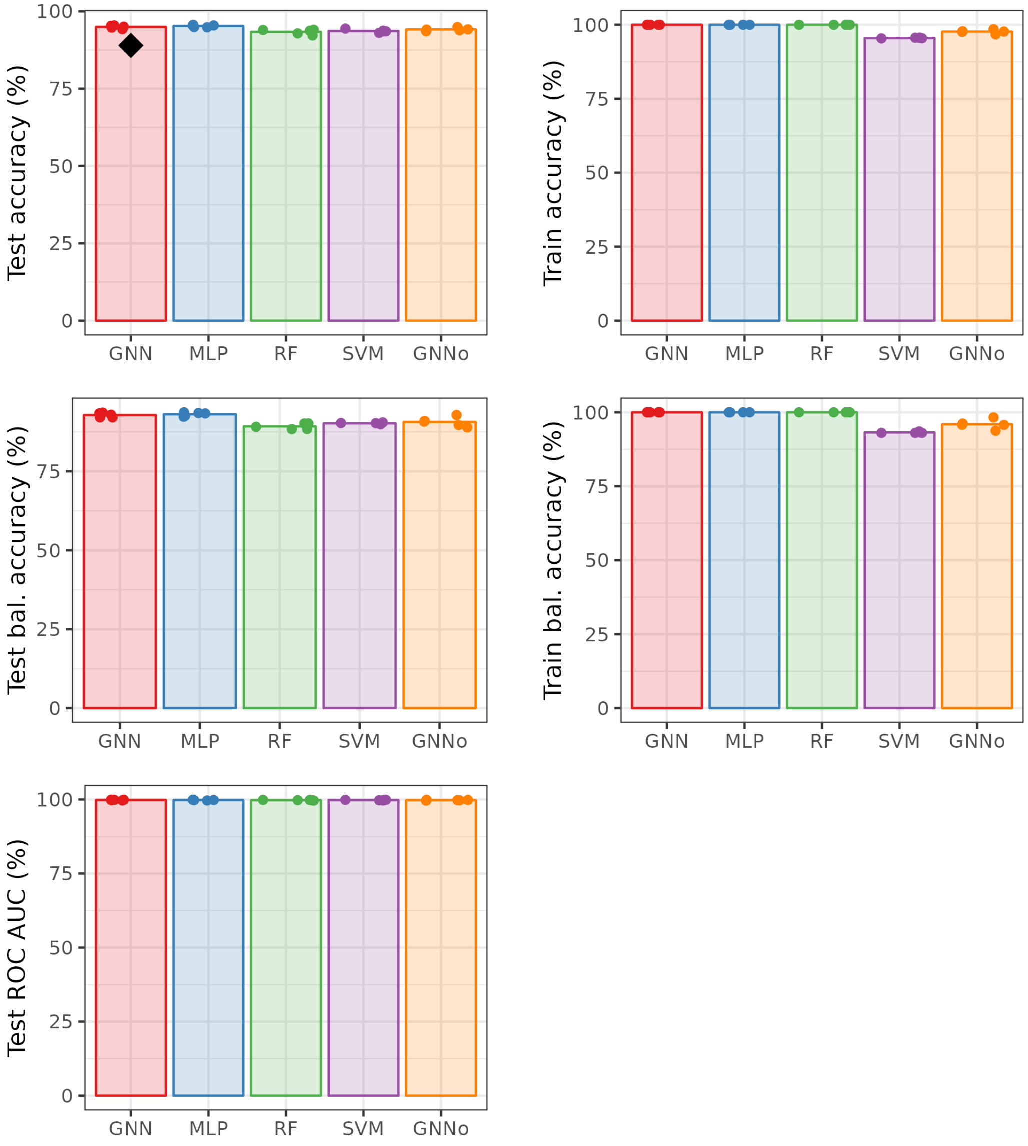

Figure S13: **CancerType.** PPI data: CV (left) and train (right) accuracy, balanced accuracy, and AUC ROC.

3.2.5 Spearman

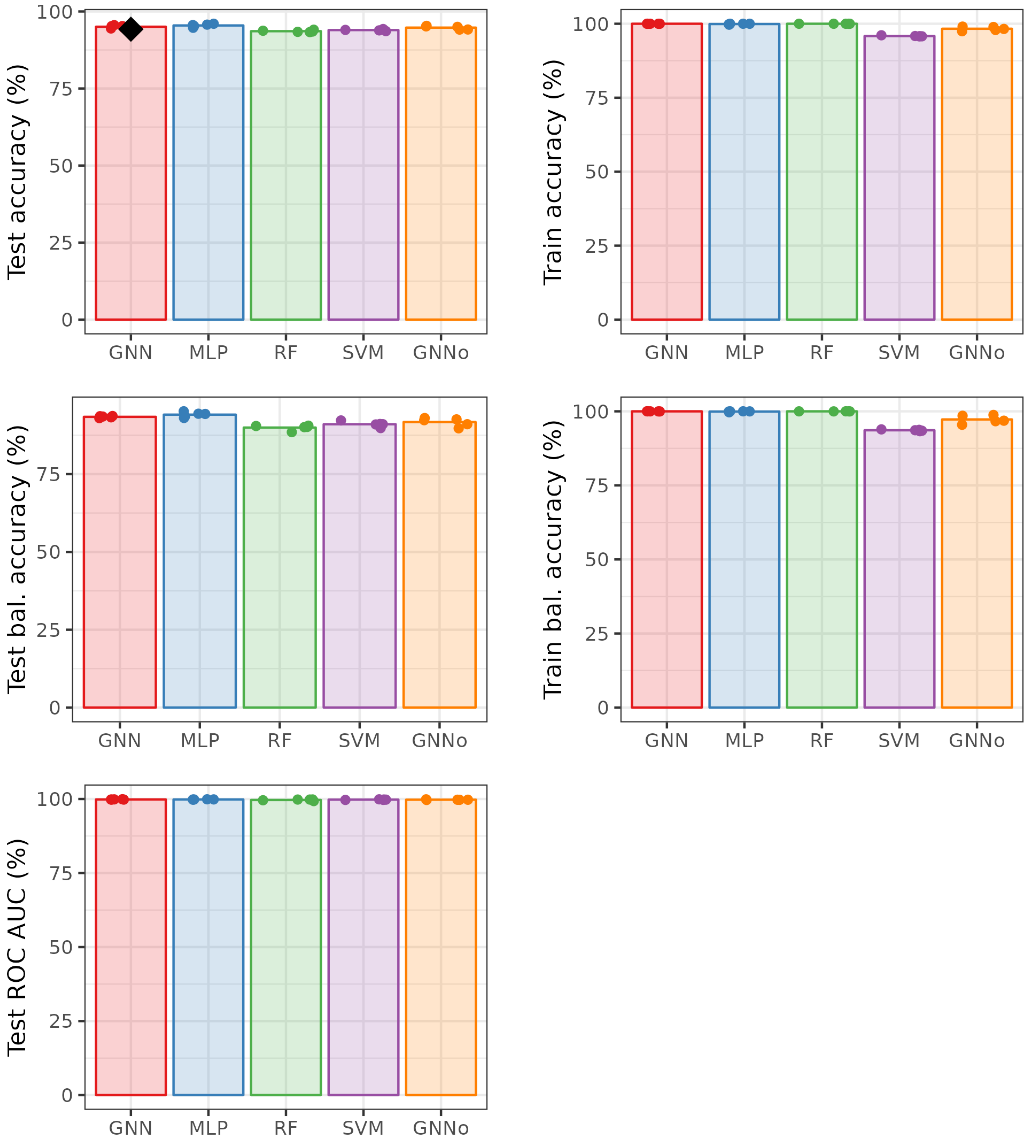

Figure S14: **CancerType.** Spearman data: CV (left) and train (right) accuracy, balanced accuracy, and AUC ROC.

3.2.6 Implementation Spearman

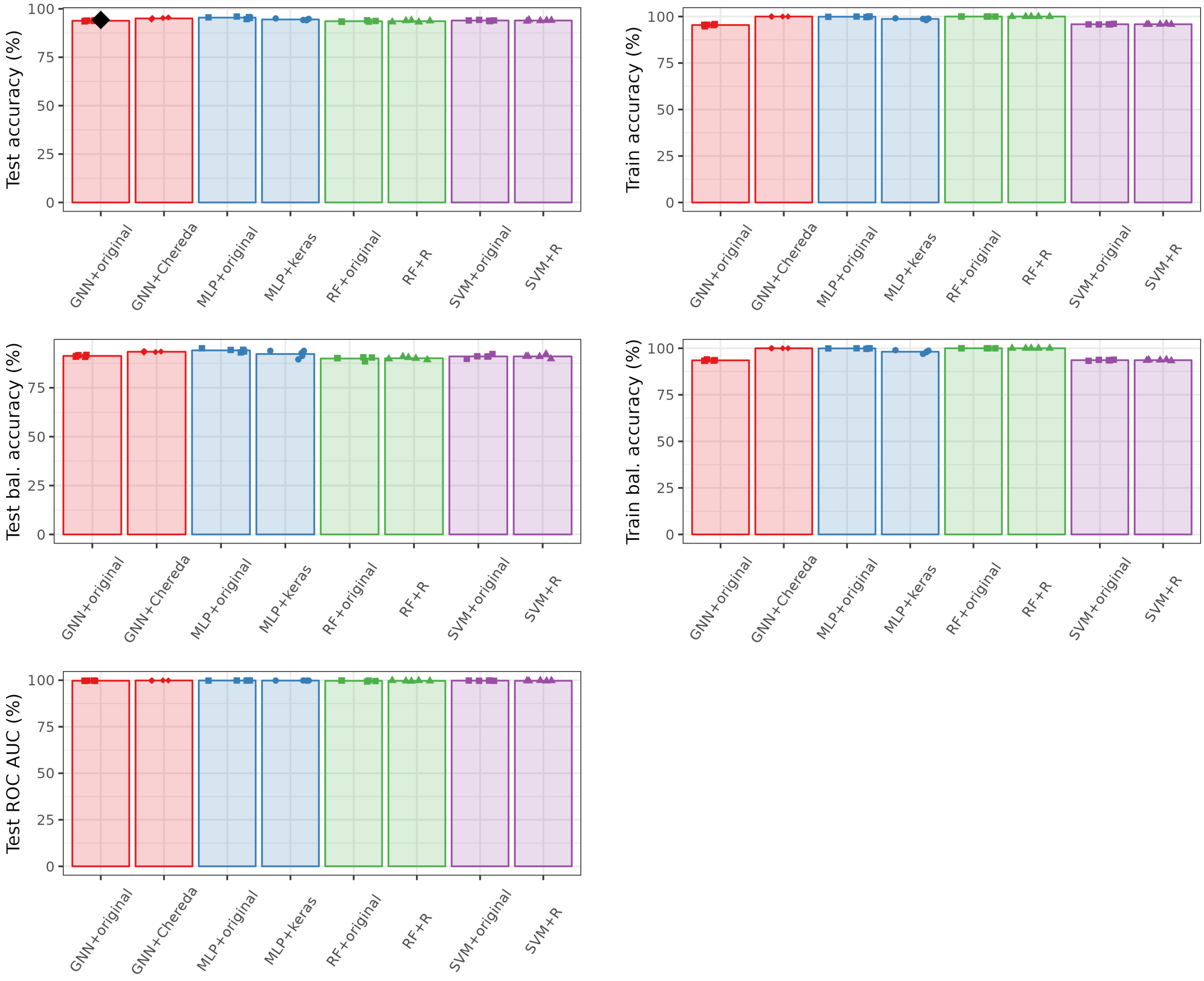

Figure S15: **CancerType.** Spearman data: CV (left) and train (right) accuracy, balanced accuracy, and AUC ROC for varying implementations.

3.3 F1000

3.3.1 Prostate

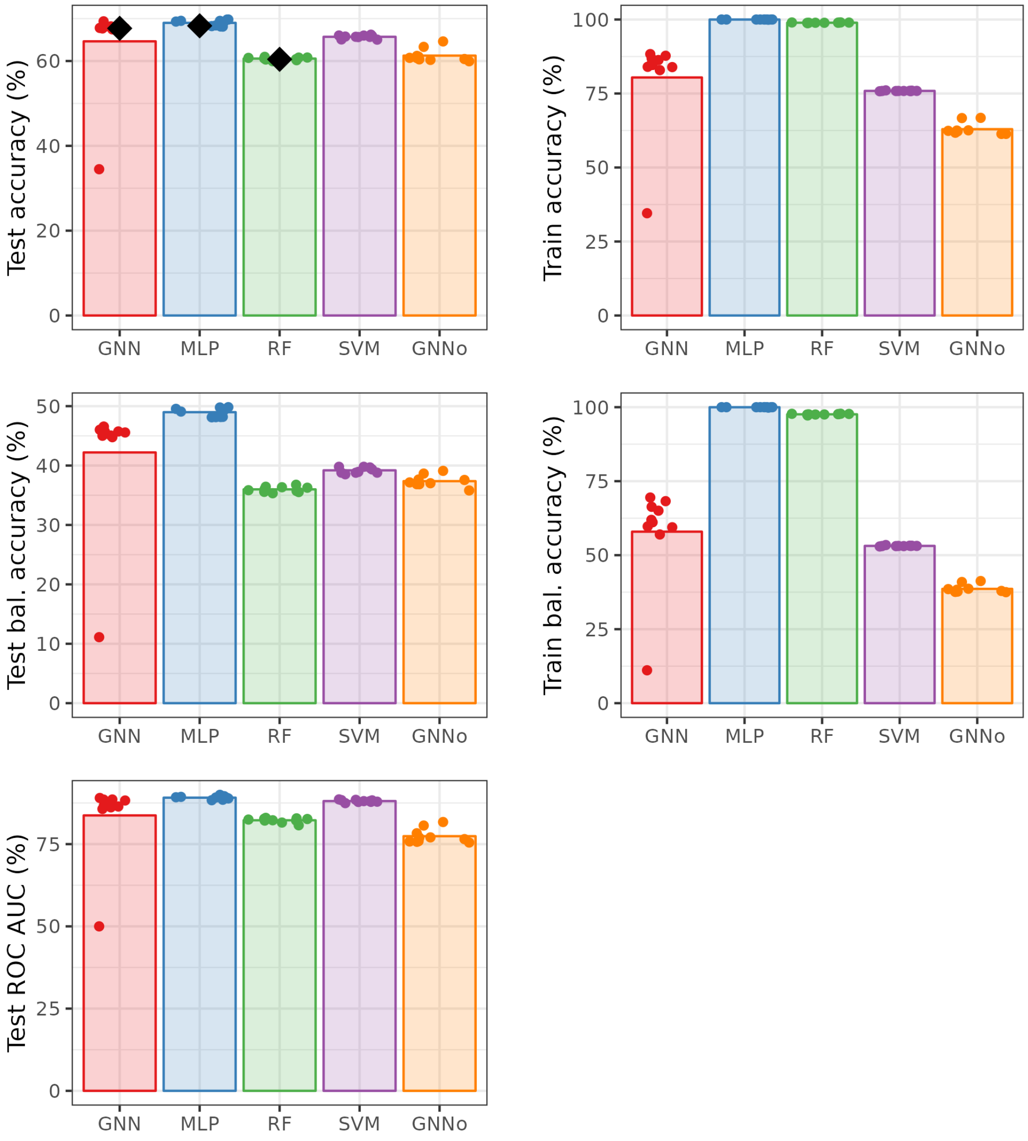

Figure S16: **F1000.** MOA data: CV (left) and train (right) accuracy, balanced accuracy, and AUC ROC.

3.3.2 Implementation (prostate)

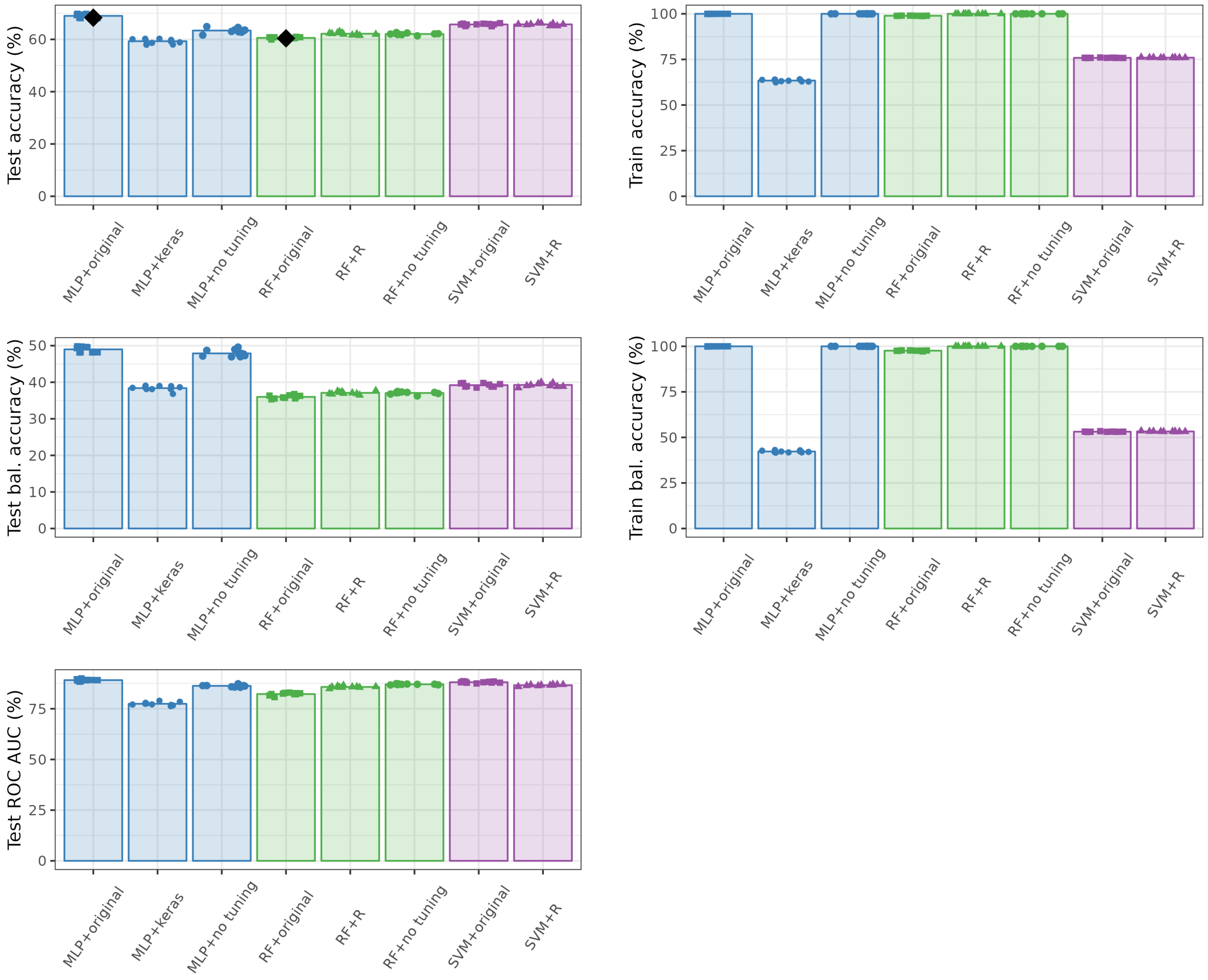

Figure S17: **F1000.** MOA data: CV (left) and train (right) accuracy, balanced accuracy, and AUC ROC for varying implementations.

3.3.3 Computational efficiency (time and memory) for prostate

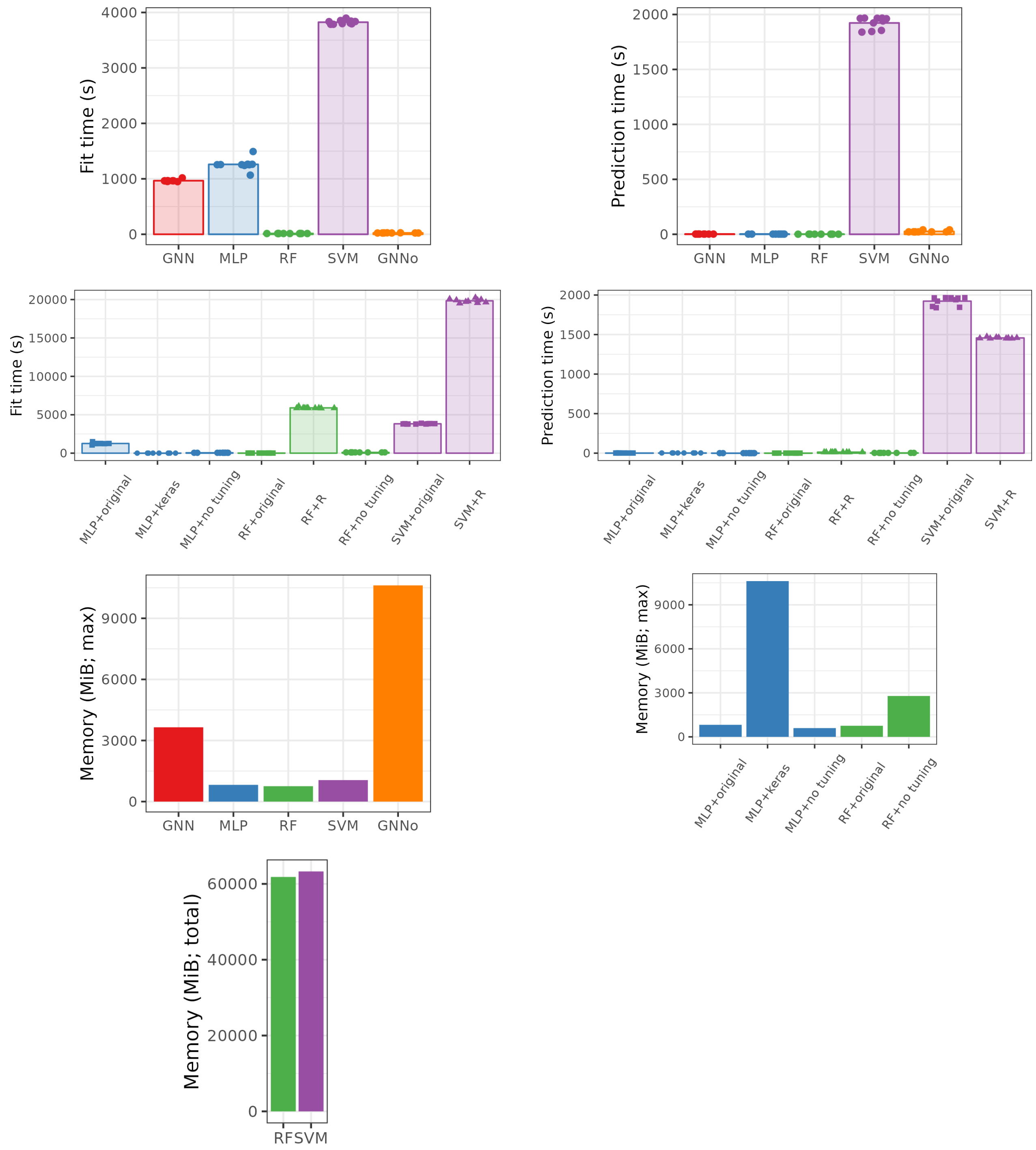

Figure S18: **F1000.** MOA data: Fit and prediction times (first row), effect of the implementation on fit and prediction times (second row), maximum memory load (in MiB) for Python scripts (third row), and total memory load (in MiB) for R scripts (fourth row).

3.3.4 Full + subtype

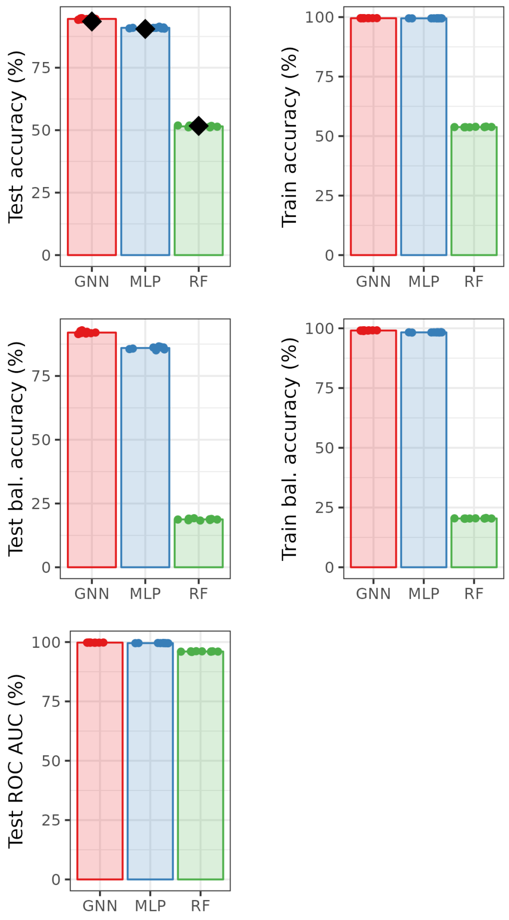

Figure S19: **F1000 full.** Subtype data: CV (left) and train (right) accuracy, balanced accuracy, and AUC ROC.

3.3.5 Implementation (full + subtype)

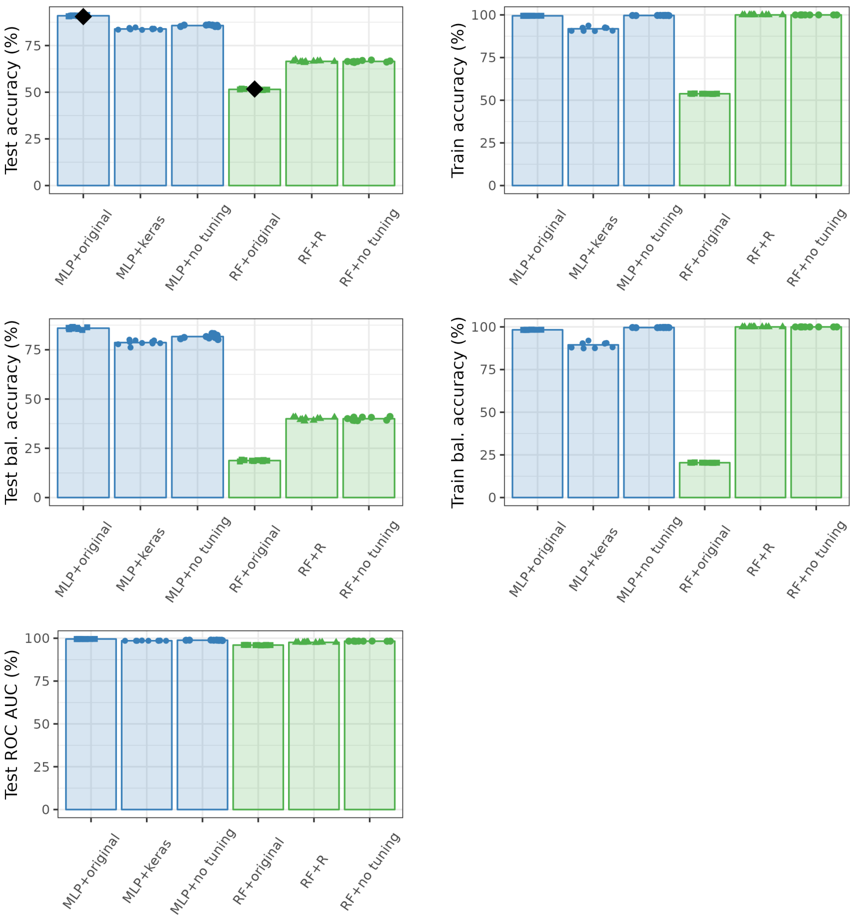

Figure S20: **F1000 full.** Subtype data: CV (left) and train (right) accuracy, balanced accuracy, and AUC ROC for varying implementations.

3.3.6 Computational efficiency (time and memory) for full + subtype

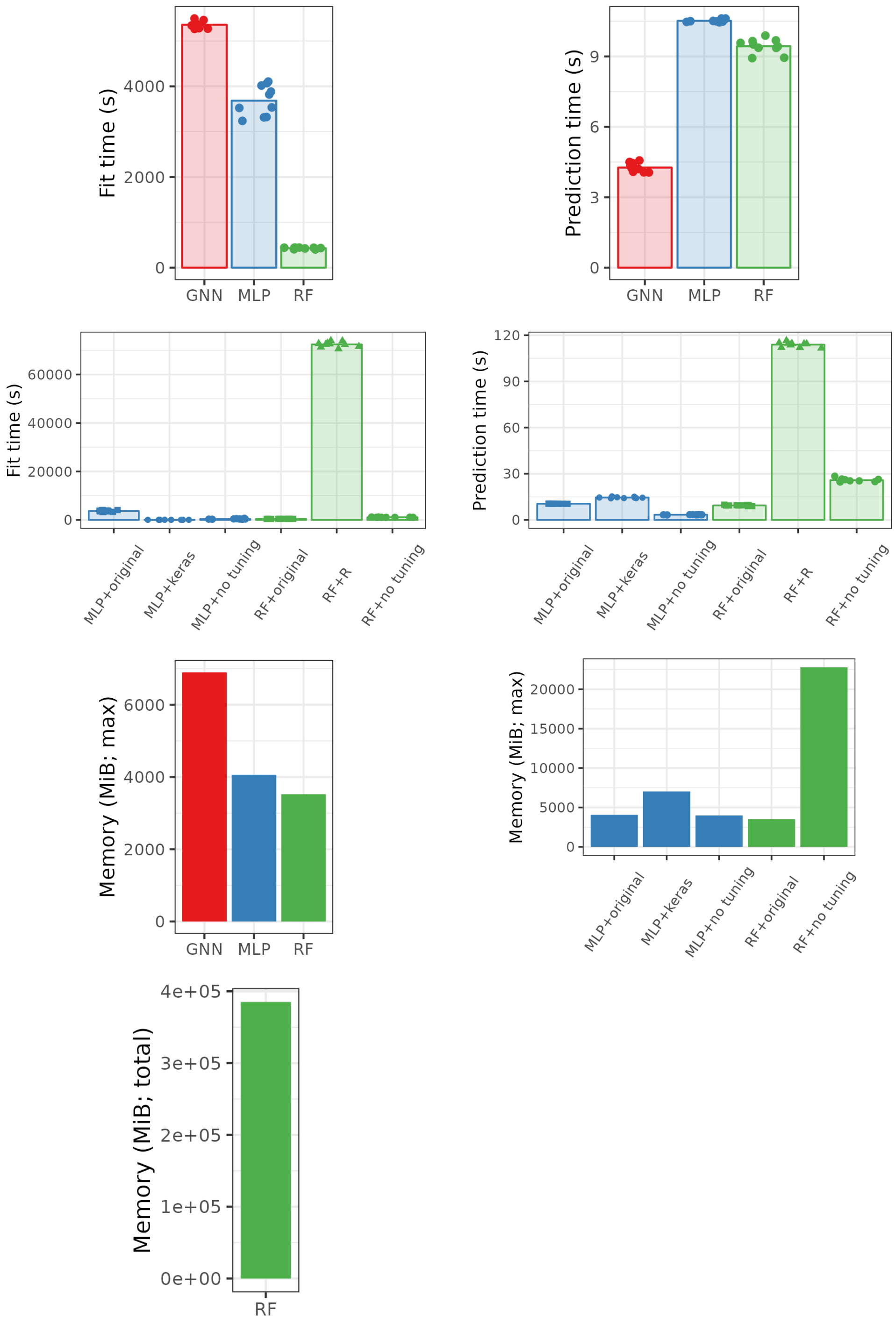

Figure S21: **F1000.** Subtype data: Fit and prediction times (first row), effect of the implementation on fit and prediction times (second row), maximum memory load (in MiB) for Python scripts (third row), and total memory load (in MiB) for R scripts (fourth row).

3.3.7 Full + primary site

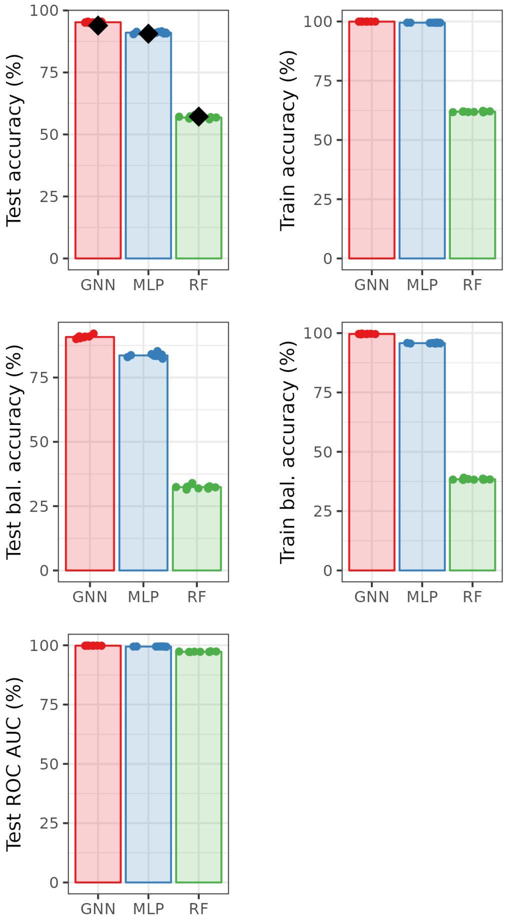

Figure S22: **F1000 full.** Primary site data: CV (left) and train (right) accuracy, balanced accuracy, and AUC ROC.

3.3.8 Implementation (full + primary site)

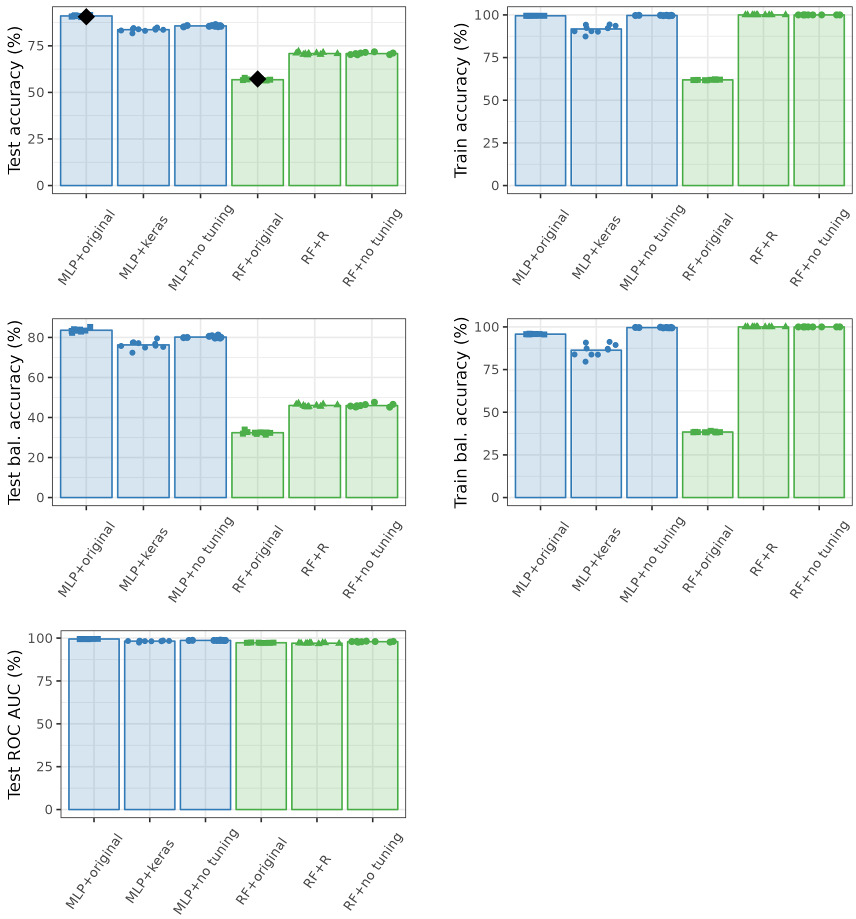

Figure S23: **F1000 full.** Primary site data: CV (left) and train (right) accuracy, balanced accuracy, and AUC ROC for varying implementations.

3.3.9 Computational efficiency (time and memory) for full + primary site

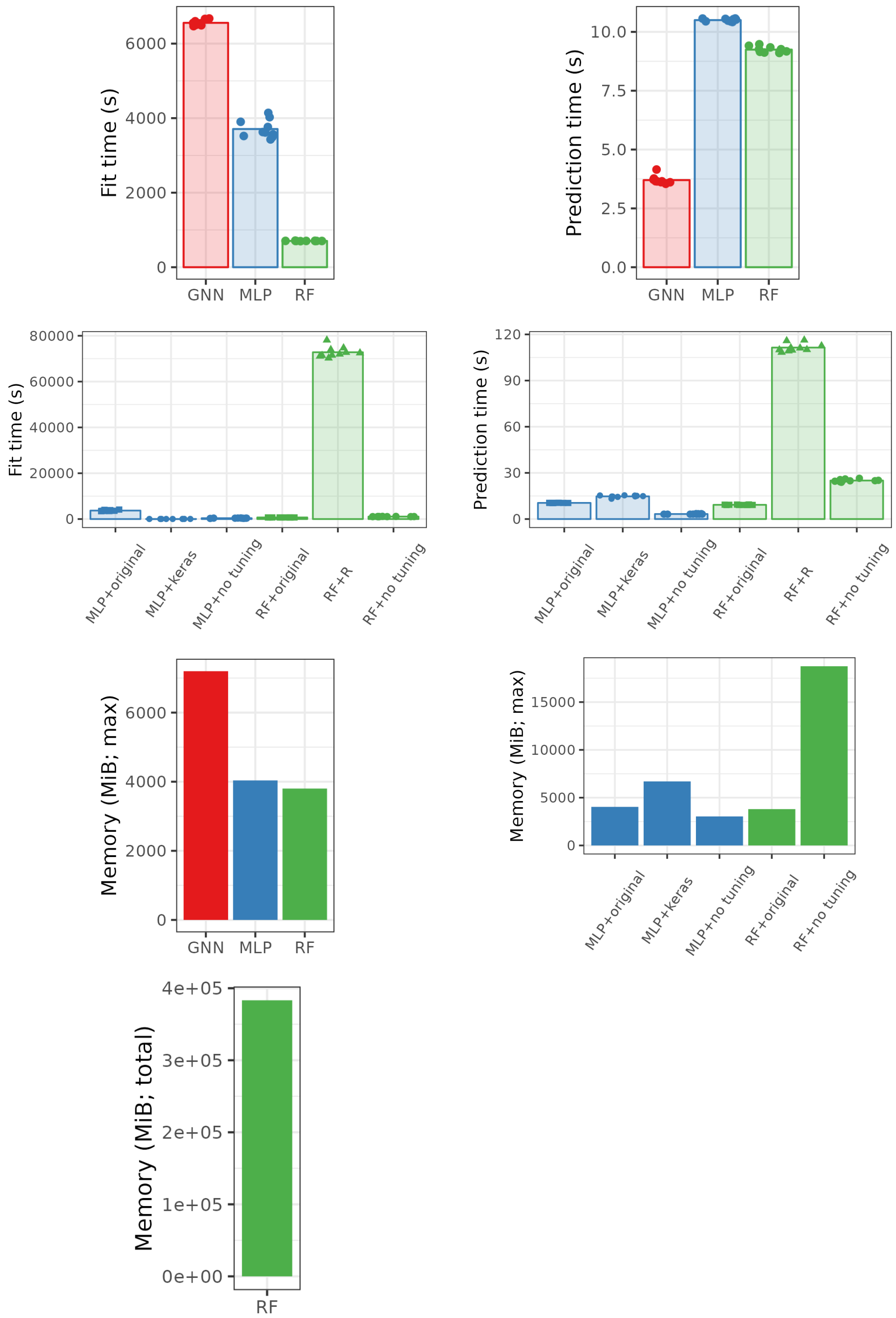

Figure S24: **F1000.** Primary site data: Fit and prediction times (first row), effect of the implementation on fit and prediction times (second row), maximum memory load (in MiB) for Python scripts (third row), and total memory load (in MiB) for R scripts (fourth row).

3.3.10 Full + MOA

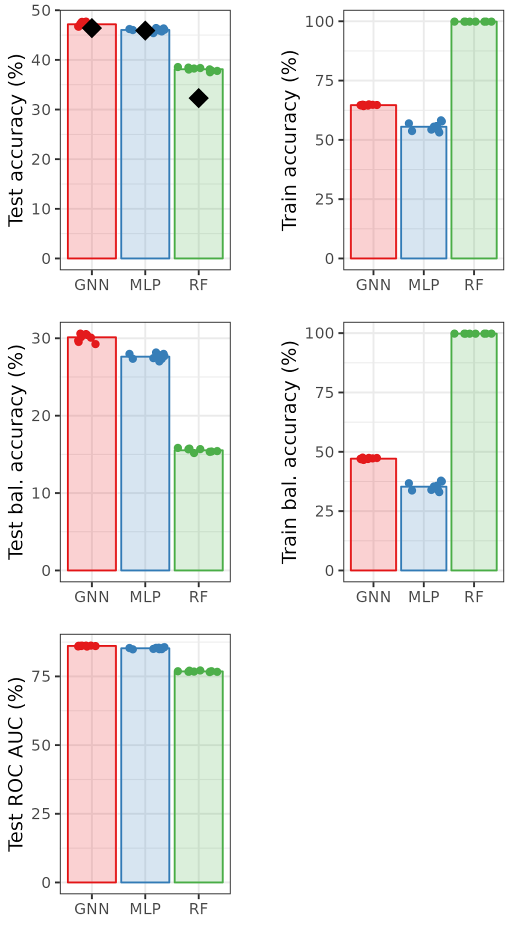

Figure S25: **F1000 full.** MOA data: CV (left) and train (right) accuracy, balanced accuracy, and AUC ROC.

3.3.11 Implementation (full + MOA)

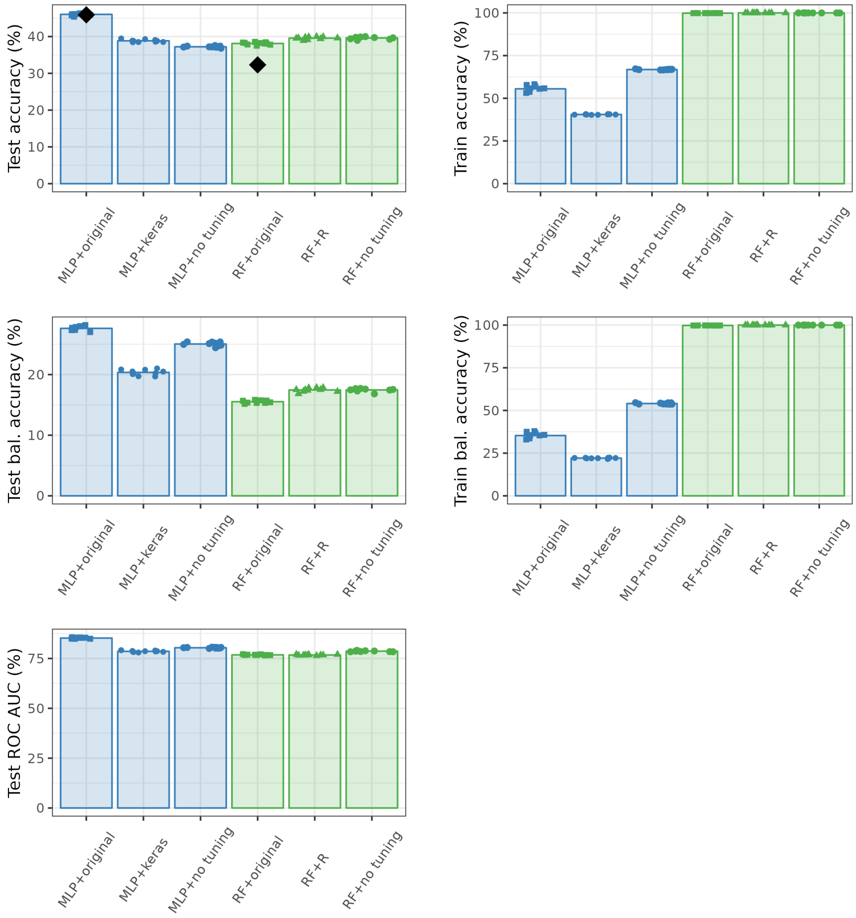

Figure S26: **F1000 full.** MOA data: CV (left) and train (right) accuracy, balanced accuracy, and AUC ROC for varying implementations.

3.3.12 Computational efficiency (time and memory) for full + MOA

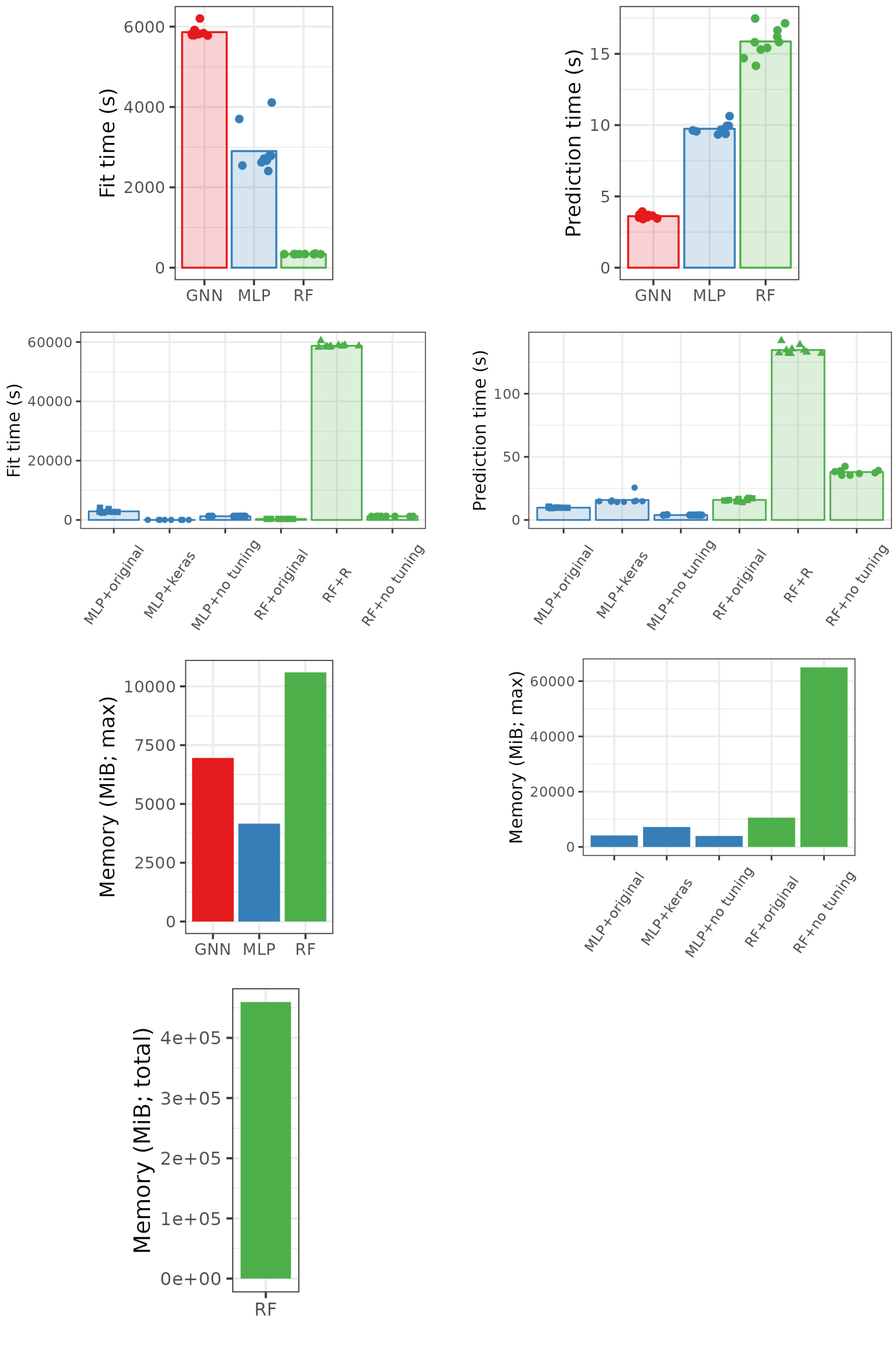

Figure S27: **F1000.** MOA data: Fit and prediction times (first row), effect of the implementation on fit and prediction times (second row), maximum memory load (in MiB) for Python scripts (third row), and total memory load (in MiB) for R scripts (fourth row).

3.4 Simulated (sismonr)

3.4.1 Methods comparison

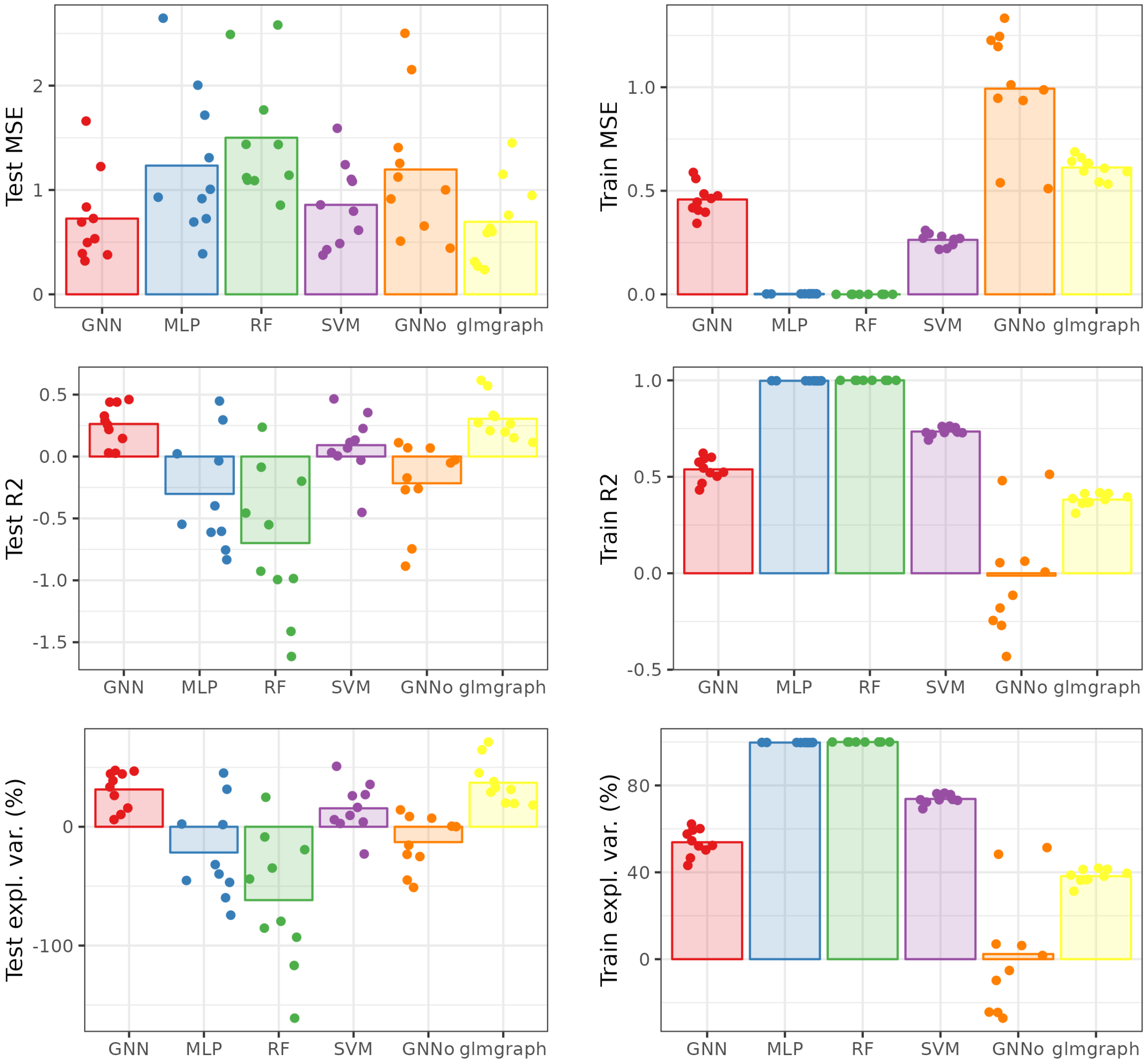

Figure S28: **Simulated.** CV (left) and train (right) mean squared error, R^2 score, and explained variance.

3.4.2 Implementation

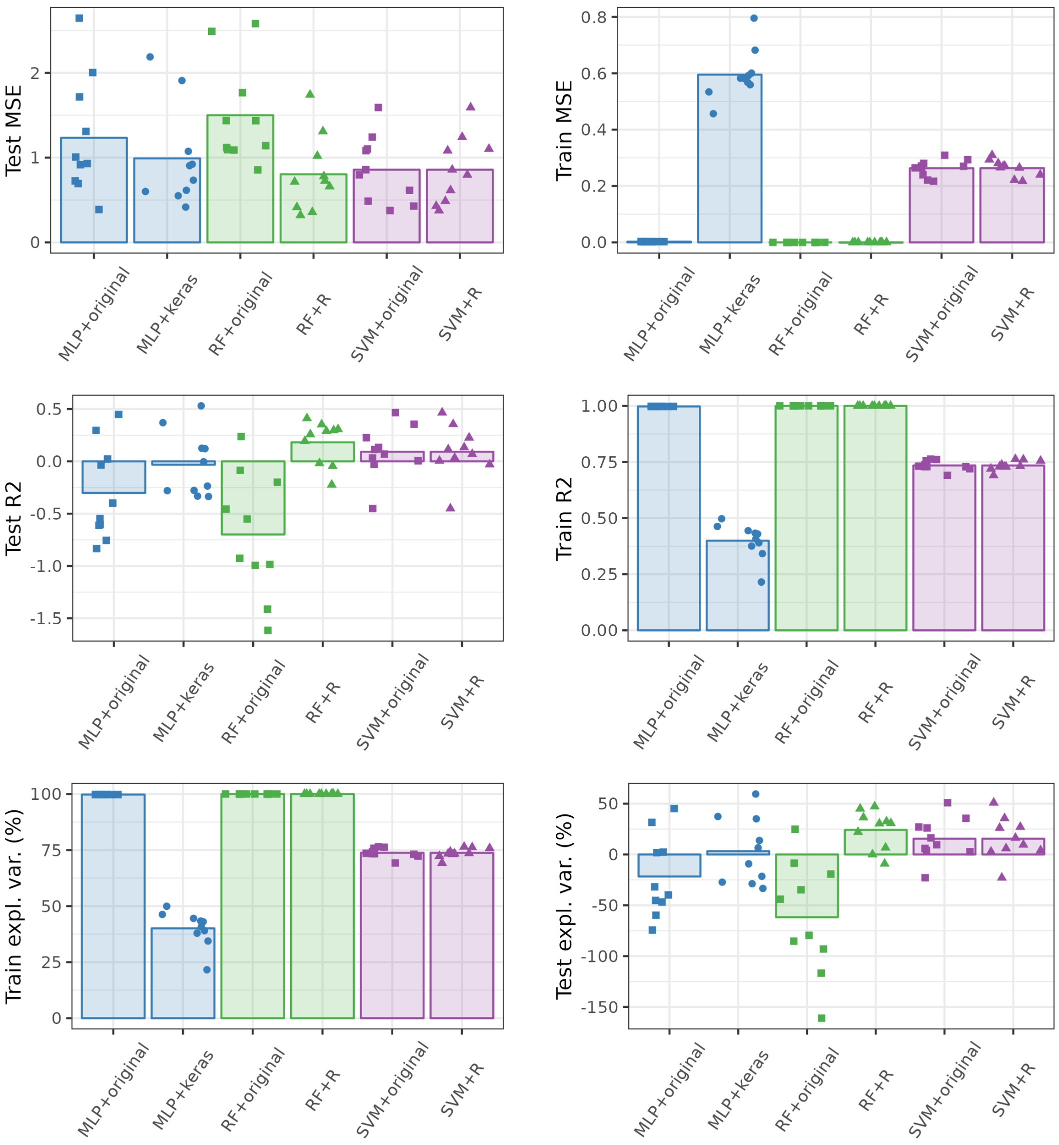

Figure S29: **Simulated.** CV (left) and train (right) mean squared error, R^2 score, and explained variance for varying implementations.

3.4.3 Graphs

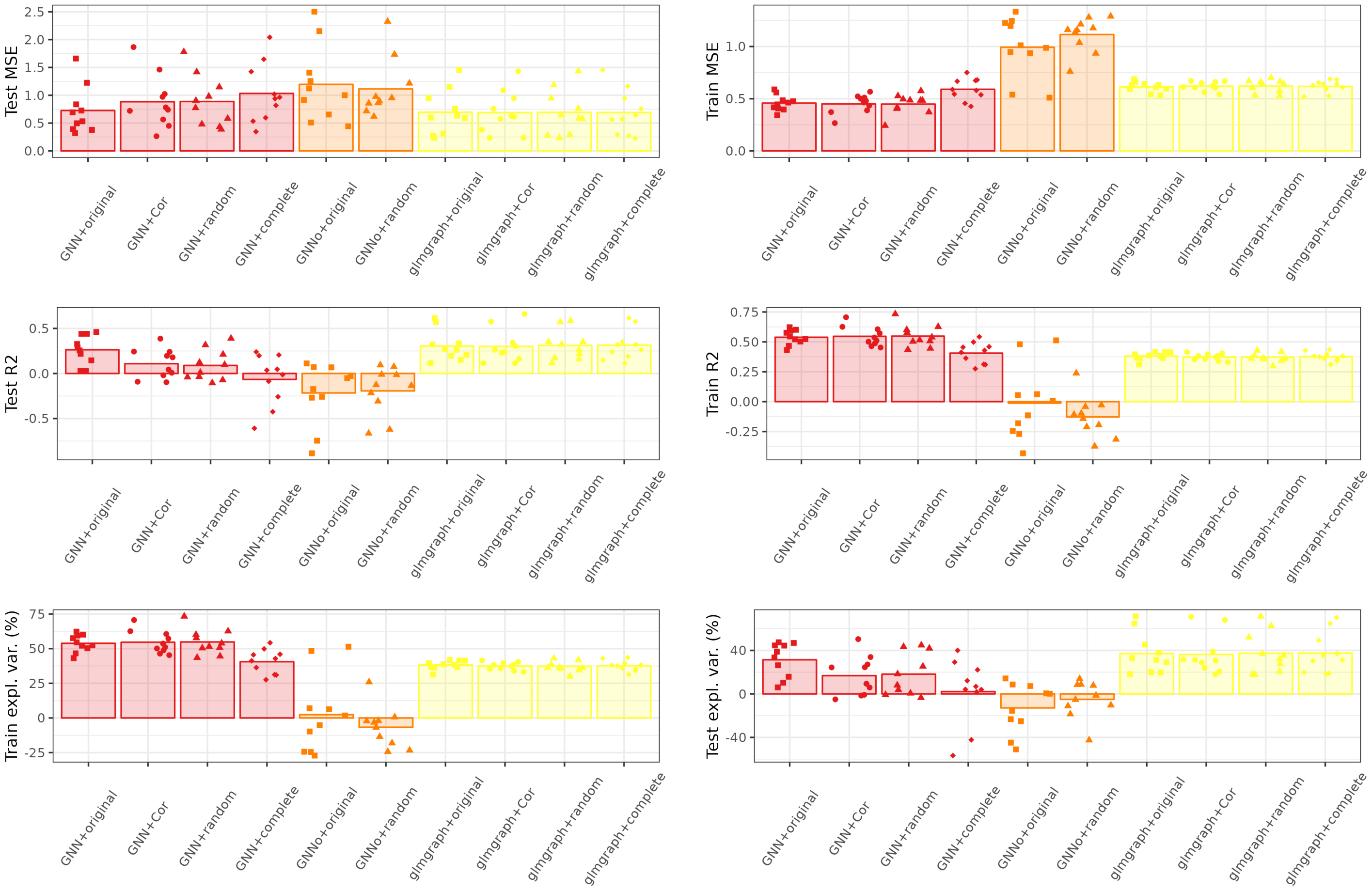

Figure S30: **Simulated.** CV (left) and train (right) mean squared error, R^2 score, and explained variance for varying input graphs.

3.4.4 Computational efficiency (time and memory)

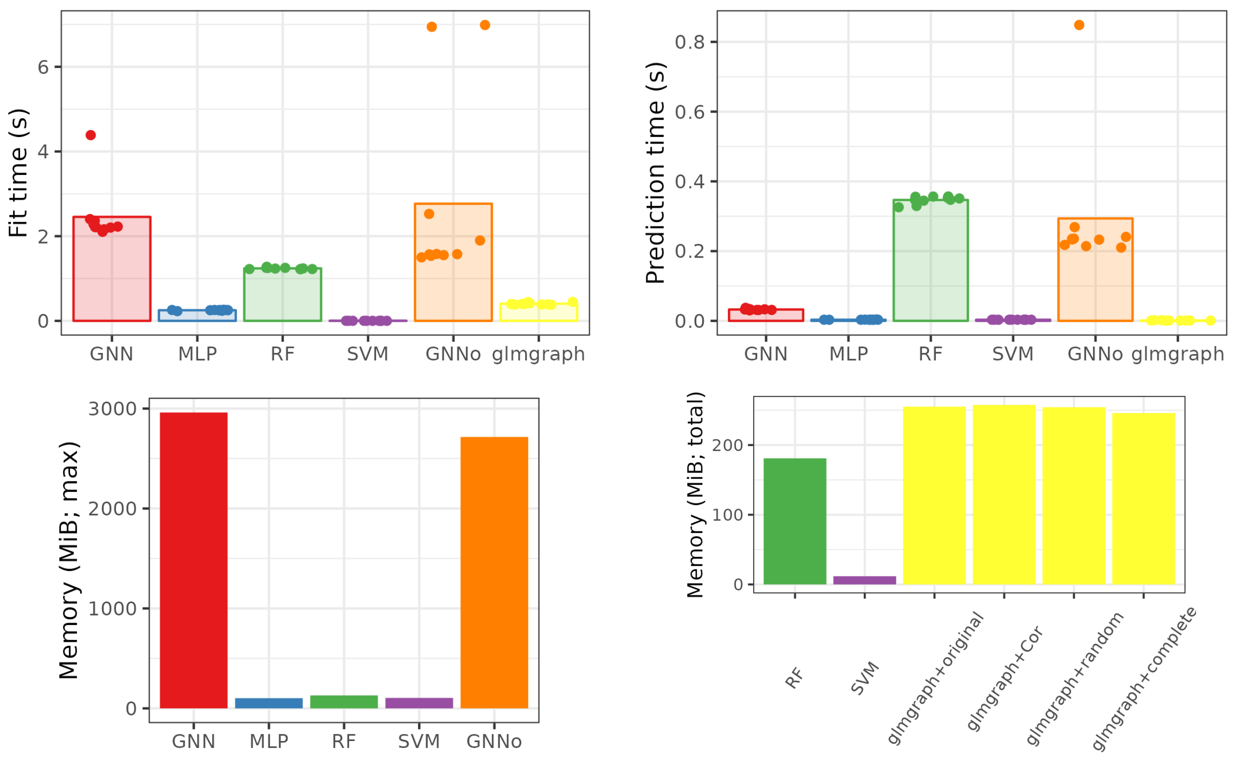

Figure S31: **Simulated.** Fit and prediction times (first row) and maximum or total memory load (in MiB), respectively for Python scripts (left) and R scripts (right, with varying input graphs).

3.4.5 Effect of implementation and of the graph on computational efficiency

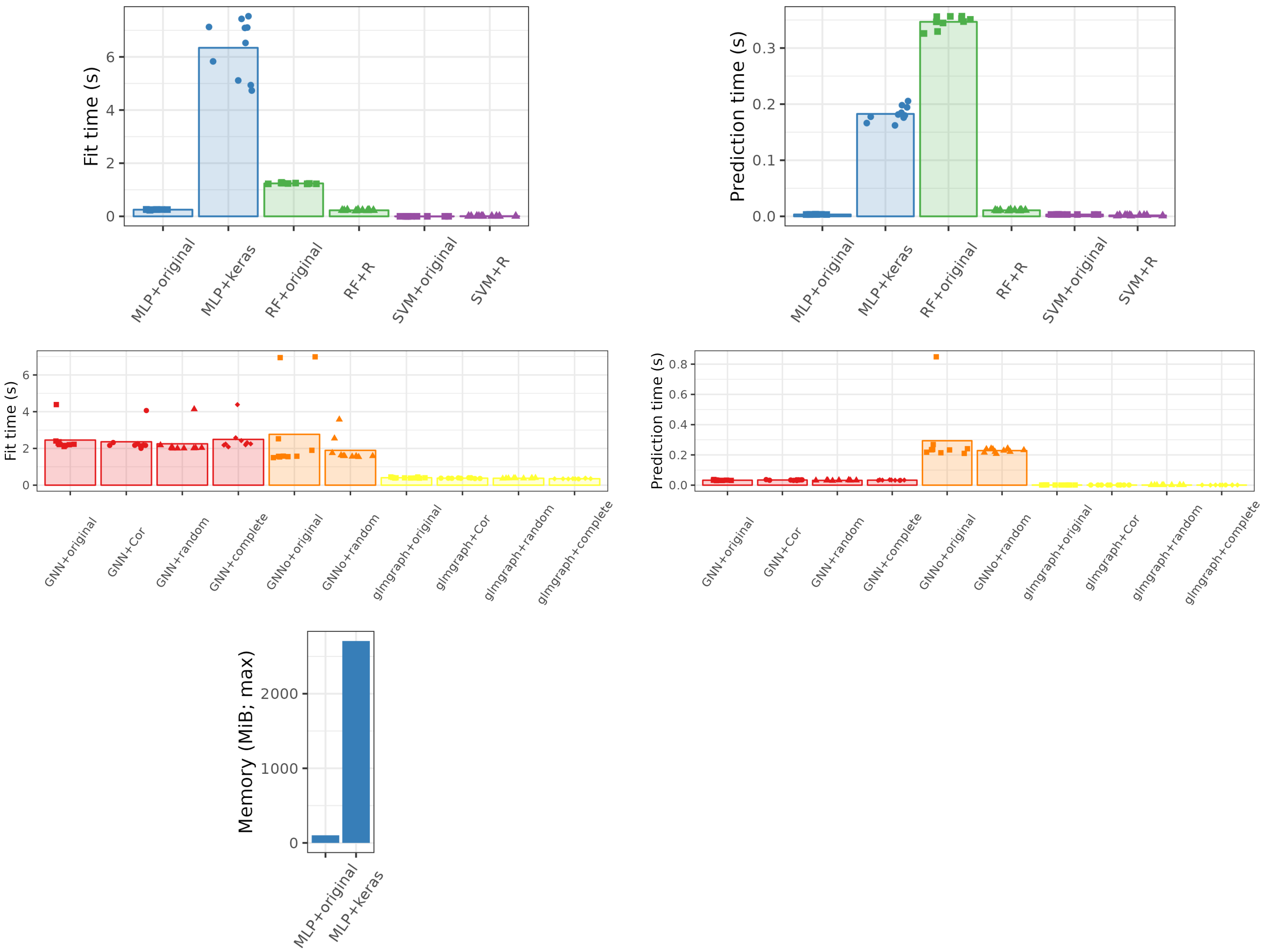

Figure S32: **Simulated.** Fit and prediction times for varying implementations (first row) and varying graphs (second row). Maximum memory load (in MiB) for Python scripts (third row).

3.5 Simulated (DREAM5, scaled data)

3.5.1 Methods comparison

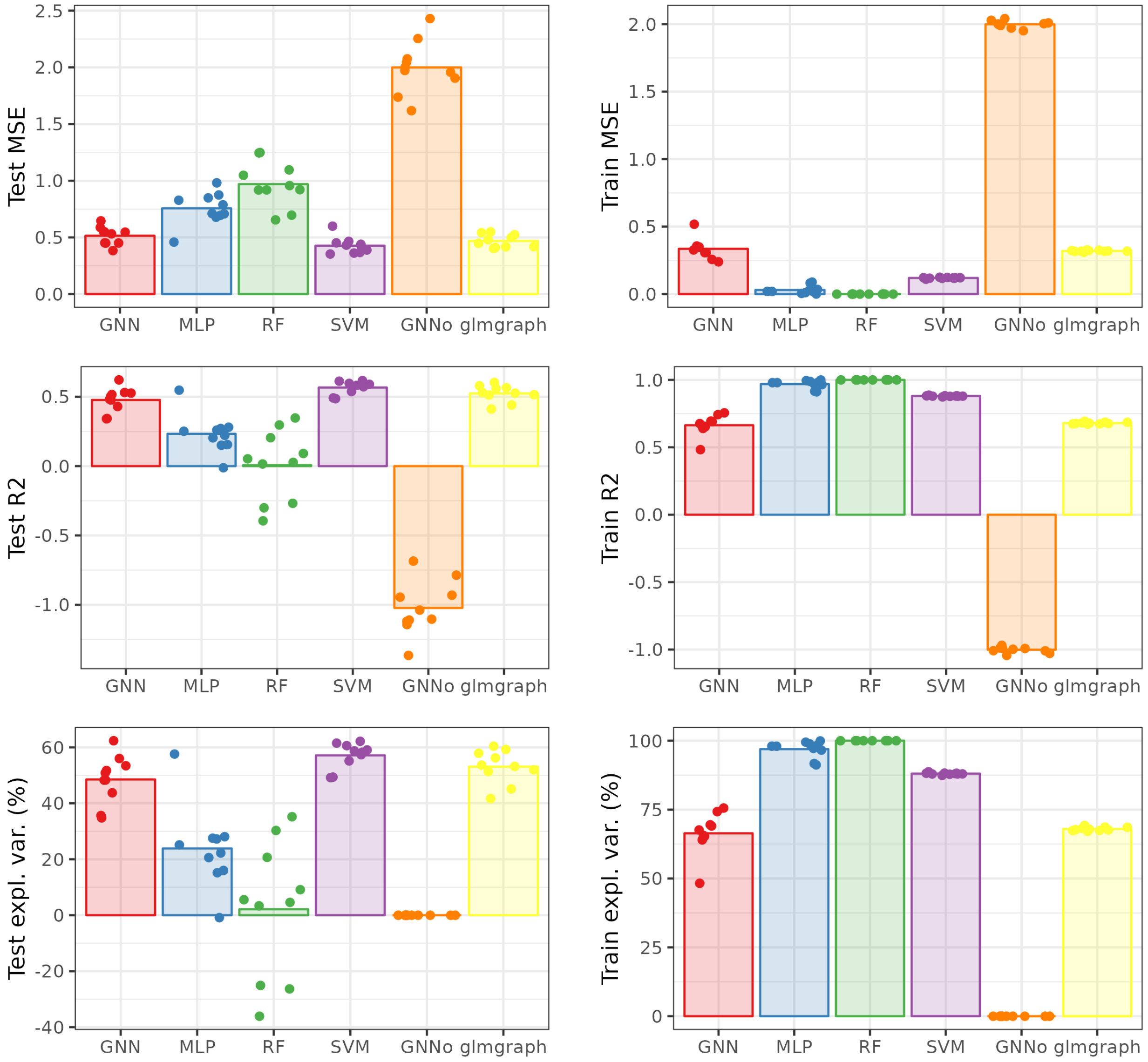

Figure S33: **DREAM5.** Scaled data: CV (left) and train (right) mean squared error, R^2 score, and explained variance.

3.5.2 Implementation

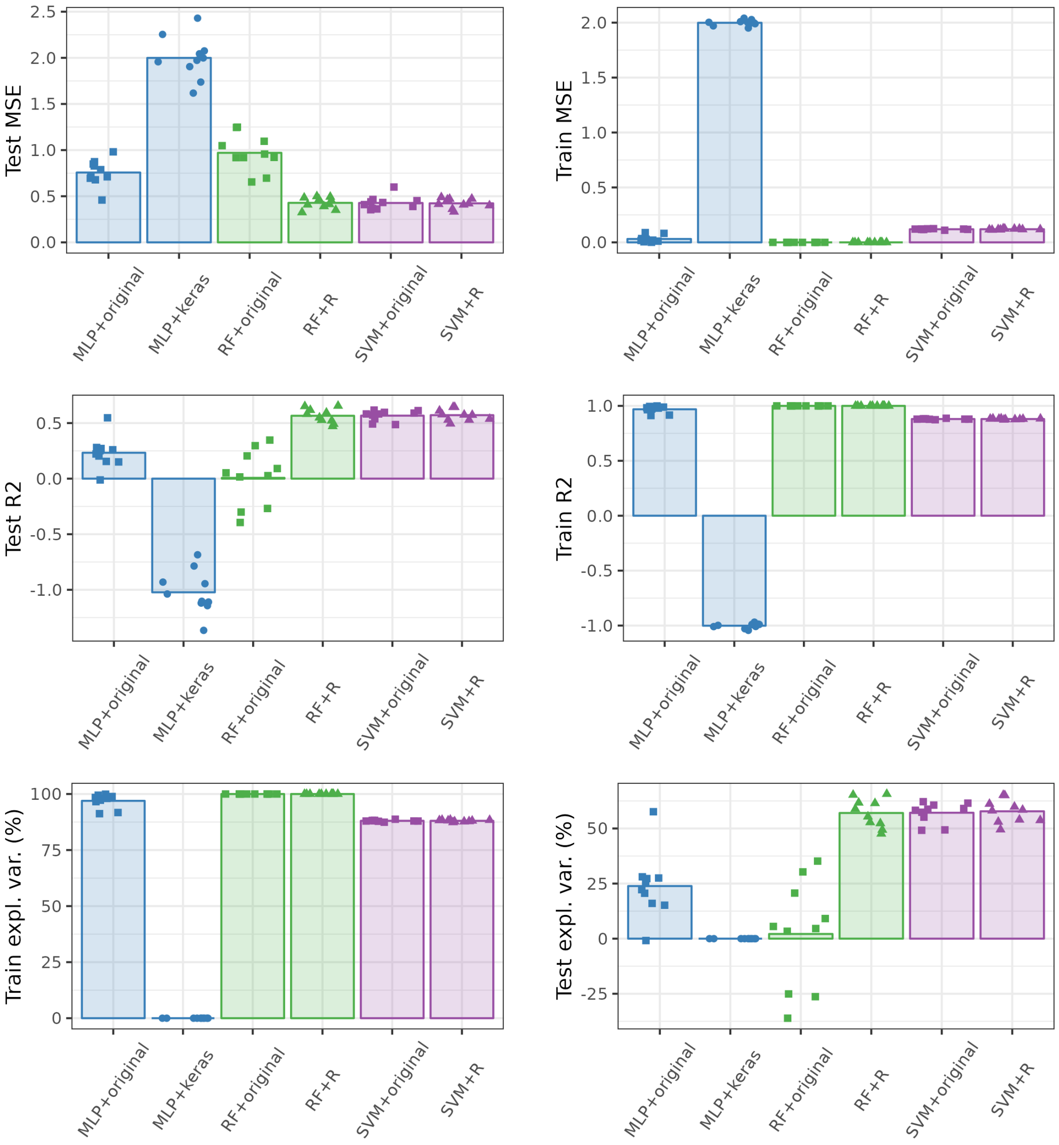

Figure S34: **DREAM5.** Scaled data: CV (left) and train (right) mean squared error, R^2 score, and explained variance for varying implementations.

3.5.3 Computational efficiency (time and memory)

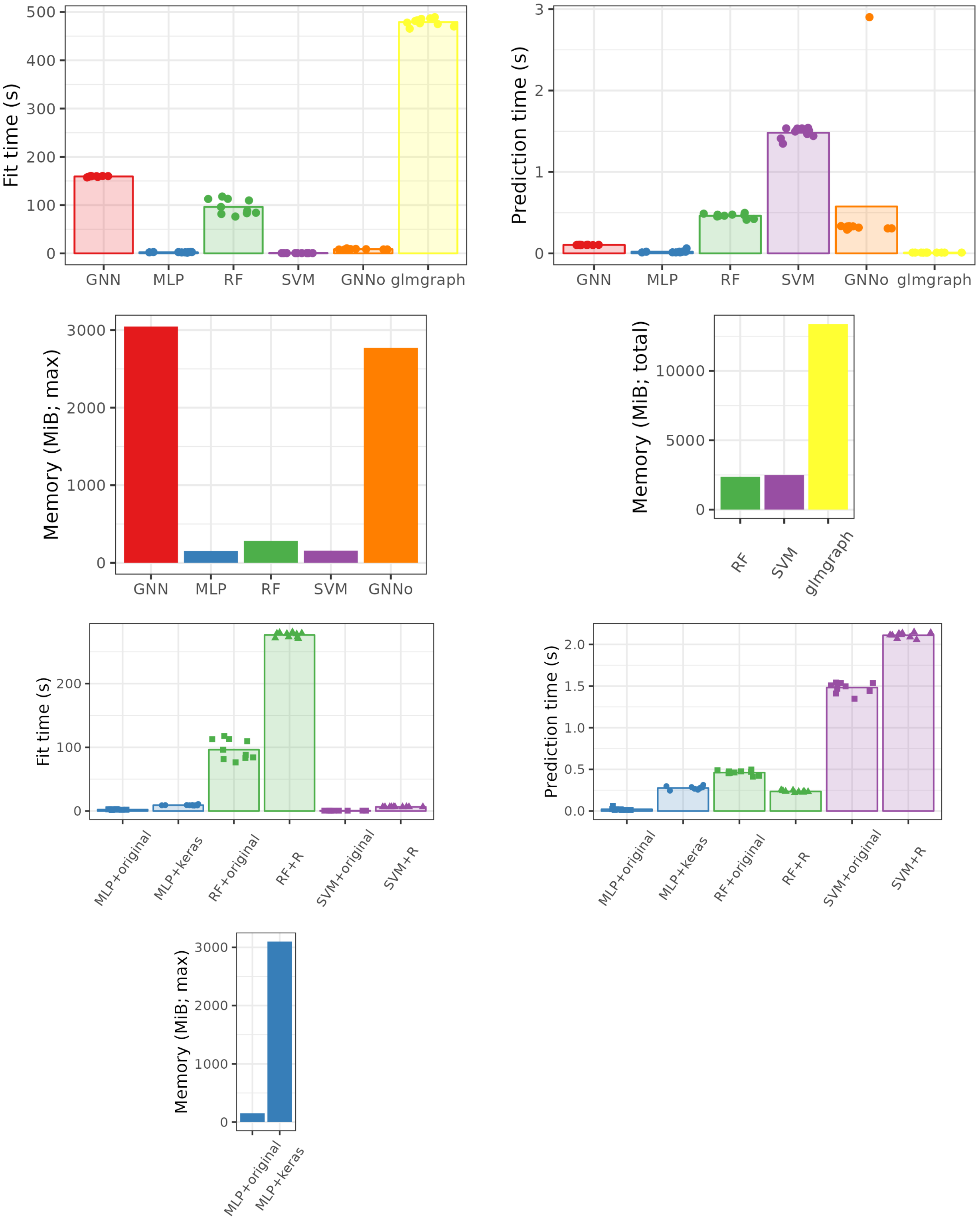

Figure S35: **DREAM5.** Scaled data: Fit and prediction times (first row), maximum or total memory load (second row; in MiB), respectively for Python scripts (left) and R scripts (right), effect of the implementation on fit and prediction times (third row), and on the maximum memory load for Python scripts (fourth row).

References

[1] Hryhorii Chereda, Annalen Bleckmann, Kerstin Menck, Júlia Perera-Bel, Philip Stegmaier, Florian Auer, Frank Kramer, Andreas Leha, and Tim Beißbarth. Explaining decisions of graph convolutional neural networks: patient-specific molecular subnetworks responsible for metastasis prediction in breast cancer. *Genome Medicine*, 13:42, 2021.

[2] Hryhorii Chereda, Annalen Bleckmann, Frank Kramer, Andreas Leha, and Tim Beissbarth. Utilizing molecular network information via graph convolutional neural networks to predict metastatic event in breast cancer. *Studies in Health Technology and Informatics*, 267:181–186, 2019.

[3] Ricardo Ramirez, Yu-Chiao Chiu, Allen Hererra, Milad Mostavi, Joshua Ramirez, Yidong Chen, Yufei Huang, and Yu-Fang Jin. Classification of cancer types using graph convolutional neural networks. *Frontiers in Physics*, 8, 2020.

[4] Matthew B.A. McDermott, Jennifer Wang, Wen-Ning Zhao, Steven D. Sheridan, Peter Szolovits, Isaac Kohane, Stephen J. Haggarty, and Roy H. Perlis. Deep learning benchmarks on L1000 gene expression data. *IEEE/ACM Transations on Computational Biology and Bioinformics*, 17(6):1846–1857, 2020.
